# Objective Tongue-Function Outcomes After Lingual Frenotomy with Adjunctive Myofascial Rehabilitation: A Retrospective Observational Longitudinal Study

**DOI:** 10.3390/jcm15135171

**Published:** 2026-07-02

**Authors:** Monika Izabela Ośko, Mira Rządzka, Arleta Czuchryta-Kamińska, Marcin Mikulewicz, Maria Cristina Manzanares Céspedes, Meritxell Sánchez Molins

**Affiliations:** 1 Odonto-Stomatology Department, University of Barcelona, 08907 Barcelona, Spain; 2 Faculty of Health Sciences, Wrocław Medical University, 50-367 Wrocław, Poland; 3 Speech Therapy Program, Faculty of Pedagogy, DSW Ideis University, 53-611 Wrocław, Poland; 4 Department of Dentofacial Orthopaedics and Orthodontics, Division of Facial Abnormalities, Wrocław Medical University, 50-367 Wrocław, Poland; 5 Human Anatomy and Embryology Unit, Experimental Pathology and Therapeutics Department, University of Barcelona, 08907 Barcelona, Spain; 6 Human Anatomy and Embryology Unit, Odonto-Stomatology Department, University of Barcelona, 08007 Barcelona, Spain

**Keywords:** ankyloglossia, lingual frenotomy, myofascial rehabilitation, oral function, tongue mobility, tongue pressure, Iowa Oral Performance Instrument, orthodontics

## Abstract

**Background/Objectives**: Restricted tongue mobility may require lingual frenotomy; postoperative scarring can cause secondary restriction, making rehabilitation relevant. We evaluated tongue mobility and pressure after frenotomy with adjunctive myofascial rehabilitation, and their association with adherence. **Methods**: This retrospective observational longitudinal study analyzed anonymized records of 64 patients aged 5–46 years treated between 2021 and 2024. All underwent frenotomy followed by a structured Myofascial Release Technique (MRT) protocol. Tongue mobility (Tongue Elevation at Maximal Mouth Opening, TEMMO) and tongue pressure (Iowa Oral Performance Instrument, IOPI) were assessed at baseline and at up to four follow-up visits (≈15 months). Adherence was classified retrospectively as high (≥3 home MRT sessions/week) or low. **Results**: Among V4 completers (*n* = 30), mean IOPI increased from 35.4 to 49.0 kPa and mean TEMMO improved from 3.1 to 1.4; 66.7% achieved TEMMO grade 1. Higher adherence was associated with better final tongue pressure (52.7 vs. 43.5 kPa; mean difference, 9.2 kPa; 95% CI, 0.8–17.6; *p* = 0.032). No postoperative complications were documented. **Conclusions**: Lingual frenotomy with adjunctive MRT-based rehabilitation was associated with favorable tongue-function changes and better adherence with better long-term outcomes among completers. Prospective controlled studies are needed to clarify the contributions of surgery, rehabilitation, and adherence.

## 1. Introduction

Tongue growth is not uniform throughout childhood. The anterior region approaches its mature dimensions earlier, generally during the first decade of life, whereas enlargement of the posterior part may continue into adolescence [1]. Embryologically, the tongue and the tissues forming the floor of the mouth initially develop as a closely integrated unit. Subsequent tissue separation contributes to the formation of the lingual frenulum and the surrounding fascial and mucosal structures [2]. Consequently, functional tongue movement depends not only on overall anatomical development but also on the mechanical freedom provided by the sublingual tissues.

Restricted tongue mobility may impair orofacial function and may contribute to difficulties in breastfeeding, swallowing, speech articulation, oral motor control, craniofacial growth, and the development or persistence of malocclusion. Ankyloglossia is most commonly considered to be a congenital condition involving a restrictive lingual frenulum, although acquired restriction related to postoperative or post-traumatic scarring may also occur [3]. Despite several anatomical and functional classification systems, no single diagnostic framework for ankyloglossia has gained universal acceptance [4].

In recent years, clinical interest in ankyloglossia and lingual frenotomy has increased substantially, together with the number of procedures performed in several countries. This has reinforced the need for objective functional assessment, especially beyond infancy, where the clinical relevance of tongue restriction should be evaluated in relation to speech, swallowing, breathing patterns, orofacial function, and orthodontic findings [5]. Although the primary aim of frenotomy is to improve tongue mobility, postoperative connective-tissue healing inevitably leads to some degree of scar formation, which may adversely affect the mechanical properties of the soft tissues surrounding the tongue [6,7].

Following injury, oral soft tissues may heal through fibrotic remodeling rather than complete regeneration. Postoperative management should not only relieve the restriction but also support functional remodeling while minimizing maladaptive scar formation [8,9].

Instrument-assisted soft-tissue mobilization (IASTM) applies controlled mechanical stimuli to promote soft-tissue adaptation. Experimental and clinical studies suggest that such stimuli may influence fibroblast activity, angiogenesis, and connective-tissue organization [10,11,12,13]. However, conventional IASTM techniques are generally applied to extraoral musculoskeletal tissues and cannot be directly transferred to the sublingual region without adaptation to the anatomical and functional conditions of the oral cavity.

Clinical observations after lingual frenotomy suggest that immediate improvement in tongue elevation may not always be fully maintained during postoperative healing. Secondary restriction may result not only from insufficient release but also from scar formation, fascial stiffness, altered motor patterns, and limited postoperative functional stimulation. This raises the need for postoperative approaches that support tissue adaptation and functional re-education while avoiding forceful stretching of the wound area.

The Myofascial Release Technique (MRT) protocol used in the present study was developed by the first author as an intraoral, instrument-assisted rehabilitation approach adapted specifically to the sublingual and perioral soft tissues after lingual frenotomy. Unlike general IASTM methods used in extraoral musculoskeletal tissues, MRT uses low-load, slow, non-painful mechanical stimulation with a dedicated intraoral instrument. The protocol is combined with functional assessment and individualized guidance addressing coexisting dysfunctions, including oral or mixed breathing and lip incompetence. Preliminary clinical observations related to this approach have been reported previously [14,15], but its association with objective long-term functional outcomes has not been systematically quantified in a large clinical sample. Postoperative rehabilitation after lingual frenotomy requires repeated patient- or caregiver-performed exercises and myofascial mobilization outside the clinical setting. In this context, therapy adherence refers to the extent to which patients or caregivers follow the recommended postoperative exercise and rehabilitation protocol, including the frequency and quality of home-based practice. This is clinically relevant because insufficient adherence may limit functional adaptation during healing and make it more difficult to interpret postoperative changes in tongue mobility and strength.

The main objective of this retrospective observational longitudinal study was to evaluate changes in tongue mobility and maximal tongue pressure in older children, adolescents, and adults undergoing lingual frenotomy followed by adjunctive myofascial rehabilitation. A secondary exploratory objective was to assess whether adherence to the postoperative rehabilitation protocol was associated with these functional outcomes over time. The manuscript first describes the clinical protocol and outcome measures, then presents longitudinal functional changes and adherence-related analyses, and finally discusses the clinical relevance and limitations of these findings.

## 2. Materials and Methods

### 2.1. Study Design and Participants

This retrospective observational longitudinal study evaluated changes in tongue-function outcomes after lingual frenotomy followed by adjunctive myofascial rehabilitation delivered according to the Myofascial Release Technique (MRT) protocol (see Section 2.4 and Supplementary File S1).

The reporting of this study was guided by the STROBE recommendations for observational research [16]. The study involved a retrospective analysis of anonymized clinical records collected from patients treated in a private dental practice providing orthodontic and myofunctional care in Poland during routine clinical care between 2021 and 2024. Because of the retrospective design, the required sample size was determined based on the number of eligible patients meeting the inclusion criteria during the study period. A reference sample-size calculation indicated that 64 patients would provide adequate statistical power for the primary endpoint (see Section 2.6).

The study consisted of 64 patients (35 females and 29 males) aged 5–46 years (median age—10 years) who were initially seen at the dental practice either after referral from external centers, predominantly by speech–language pathologists, or during orthodontic and/or speech–language consultations. All referred or self-presenting individuals underwent a standardized in-practice functional assessment based on the Multifunctional System (MFS) protocol [17,18,19] to verify the indication for frenotomy and potential adjunctive therapy. Not all referred patients met the clinical criteria for surgical management; those who did not meet the criteria did not proceed to frenotomy. The final decision regarding surgical eligibility was made by the operating dentist after standardized functional and clinical assessment, supported by postgraduate university training in advanced clinical orthodontics. The baseline demographic and clinical characteristics of the study are summarized in Table 1.

All patients underwent baseline assessment before lingual frenotomy (V0), including maximal tongue pressure measured with the Iowa Oral Performance Instrument (IOPI Model 2.3; IOPI Medical LLC, Redmond, WA, USA) [20]. Tongue mobility was evaluated with the Tongue Elevation at Maximal Mouth Opening (TEMMO) scale [17], which was derived from Durán’s original classification [18,19]. Follow-up outcome assessments were conducted immediately after surgery (V1) and at subsequent early, intermediate, and long-term postoperative visits (V2–V4). The timing of these assessments is summarized in Table 2. IOPI and TEMMO measurements were repeated at each assessment visit. Some patients also received concomitant speech–language therapy as part of routine care. The primary outcome was the change in maximal tongue pressure measured with the IOPI (kPa) from baseline (V0) to the final follow-up visit (V4). Secondary outcomes included changes in tongue mobility measured using the TEMMO assessment, the proportion of patients achieving TEMMO grade 1, and adverse events documented in the clinical records.

### 2.2. Tongue Mobility Assessment

**Figure 1:**
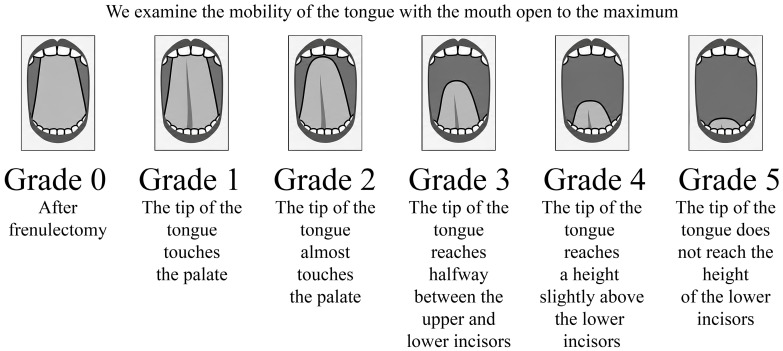
Schematic representation of the TEMMO grading system and notation. Tongue-tip elevation is assessed at maximal mouth opening: the patient is instructed to open the mouth as wide as possible and then elevate the tongue as high as possible while maintaining maximal mouth opening. In patients with a previous frenotomy, an additional “0” is recorded to indicate prior surgery; this marker does not represent a mobility grade. Current tongue mobility is reported as the final value on the 1–5 scale. For example, 0/0/3 indicates two previous frenotomies and a current mobility grade of 3.

For the present retrospective analysis, tongue mobility scores were obtained from routine clinical records. In these records, tongue elevation had been graded using the Tongue Elevation at Maximal Mouth Opening (TEMMO) scale (Figure 1). Patients were included only if their initial qualifying assessment documented restricted tongue mobility, defined as TEMMO grades 3–5. Grade 3 was used as the threshold for surgical qualification, whereas grades 4–5 were considered indicative of clinically significant ankyloglossia. TEMMO is an inverse ordinal scale: 1 indicates the best tongue elevation or full mobility, whereas 5 indicates the most restricted mobility; therefore, higher scores indicate poorer tongue mobility.

The validated/adapted TEMMO grading system used for clinical interpretation consists of ordinal mobility grades 1–5. Half-grade values are not formally validated TEMMO categories. In the original retrospective clinical records, intermediate half-grade values could be recorded when tongue elevation was judged clinically to be between two adjacent grades, for example between grades 2 and 3 after preoperative preparation but before surgery. Surgical eligibility, however, was based on the initial qualifying assessment documenting restricted tongue mobility corresponding to TEMMO grade ≥ 3. For the final analysis, half-grade values were not retained as separate analytical categories. Because TEMMO is an inverse scale, with lower grades indicating better mobility, a value such as 2.5 represents better mobility than grade 3 but does not correspond to a formally validated category. Therefore, intermediate half-grade values were conservatively assigned to the numerically higher, functionally more restricted adjacent grade for categorical analysis to avoid overestimating improvement. Consequently, Table 1 and all subsequent analyses report TEMMO using integer grades only, and the baseline TEMMO range was reported as 3–5.

Half-grade values were not used as separate validated categories in the inter-rater reliability analysis. TEMMO reliability was assessed using integer mobility grades on the 1–5 scale. To assess inter-rater reliability, TEMMO grading was independently performed by two clinicians (a speech–language therapist and the operating dentist) in a random sample of 15 patients at two assessment time points (30 ratings in total). Each clinician completed the ratings independently and had no access to the other examiner’s scores. Inter-rater agreement was assessed using Cohen’s kappa and reported with 95% confidence intervals.

### 2.3. Tongue Pressure Assessment and Clinical Outcomes

Intraoral pressure was assessed using the IOPI Model 2.3 (IOPI Medical LLC, Redmond, WA, USA), which records maximal tongue pressure in kilopascals (kPa). At each follow-up visit, tongue mobility, tongue deviation, and intraoral pressure were reassessed to document functional changes over time. Outcome measures included maximal tongue pressure measured with the IOPI, tongue mobility assessed with TEMMO, tongue deviation evaluation, range of maximum mouth opening (RMMO), and descriptive assessment of occlusal relationships, including vertical, transverse, and sagittal relationships and the presence of crossbite. RMMO was recorded in millimeters during routine clinical assessment and monitored across follow-up visits together with tongue mobility and tongue pressure. Subjective patient-reported observations regarding articulation, facial muscle tone, swallowing comfort, perceived freedom of tongue mobility, and comfort during nasal breathing were recorded during routine clinical follow-up when reported, but they were not collected using validated questionnaires and were therefore not analyzed as formal study outcomes.

### 2.4. Preoperative Preparation, Surgery, and Rehabilitation Protocol

Preoperative preparation, consisting of manual exercises or MRT-based therapy, was implemented in 48.4% of cases. This preoperative phase was individualized according to baseline anatomical restriction, TEMMO grade, tissue tolerance, previous speech–language therapy, and practical accessibility to treatment. In patients with marked ankyloglossia and strong anatomical attachment between the tongue and the floor of the mouth, forceful preoperative stimulation of the sublingual region was avoided to prevent excessive irritation or tension in the frenulum attachment area. In the full study sample, 35.9% of patients received MRT-based preoperative therapy, whereas 12.5% received manual preoperative therapy.

Preoperative preparation occurred through two pathways: patients referred through speech–language therapy services often presented after having already completed manual preoperative preparation, whereas patients without prior therapy were offered preoperative MRT-based preparation in the clinic. Some families declined preoperative preparation for logistical reasons, including long travel distance and a preference for completing the surgical step in a single visit. The remaining 51.6% did not undergo specific preoperative preparation.

All 64 patients underwent lingual frenotomy limited to the release of mucosal and fascial components, without incision of the tongue musculature. Scissors were the primary instrument in 34 patients (53.1%), and electrosurgical incision in cutting mode without additional coagulation was the primary instrument in 29 patients (45.3%); one patient (1.6%) received both techniques during the same procedure. The release was carried out through two limited incision lines rather than a single wider cut. The first incision was made in the area separating the alveolar ridge from the sublingual ductal region, whereas the second was positioned between the ductal region and the ventral attachment of the frenulum.

This approach was intended to obtain sufficient release of the restrictive mucosal–fascial component while avoiding a wide lateral wound, excessive lateral extension, deep dissection, or unnecessary thermal or mechanical injury to adjacent tissues.

Before injection, topical anesthesia with 10% lidocaine was applied to the sublingual mucosa. Local infiltrative anesthesia was then administered using a small volume of dental local anesthetic with vasoconstrictor (Dentocaine 40 mg/mL + 0.01 mg/mL, articaine hydrochloride with epinephrine/adrenaline), approximately one-third of a dental cartridge, through a single needle insertion placed several millimeters posterior to the sublingual caruncle/ductal opening.

The surgical approach was guided by recent topographic anatomical descriptions of the lingual frenulum and floor of the mouth in neonates and adults. These studies describe the lingual frenulum as a dynamic mucosal–fascial fold formed by oral mucosa and underlying floor-of-mouth fascia, rather than as an isolated midline band [6,7]. They also show that superficial branches of the lingual nerve may lie immediately deep to the fascial layer on the ventral tongue and may pass medially toward the frenulum. Therefore, a midline excision of the frenulum could theoretically transect or injure these fine neural branches. For this reason, the procedure was performed as a limited frenotomy, with two small incisions. The aim of this two-incision approach was to release the restrictive mucosal–fascial component while avoiding wide lateral extension, deep dissection, or tissue excision in areas where superficial lingual-nerve branches may be present. The submandibular duct openings and sublingual caruncles were identified visually and avoided. All procedures were performed under magnification with dental loupes and direct visual control with attention to avoiding the ductal openings, sublingual caruncles, floor-of-mouth vasculature, and superficial lingual-nerve branches. This comparable neonatal and adult topographic relationship is further illustrated in Supplementary Figure S2.

The choice between scissors and electrosurgical incision was made clinically by the operating dentist and was not based on random allocation. Electrosurgical incision was generally selected when the use of a fine active electrode allowed better visualization of the intended incision line and precise control of the release within the restrictive mucosal–fascial component. Because electrosurgery involves thermal energy, it was used cautiously and only in cutting mode without additional coagulation. Scissors were preferred in younger or less cooperative patients, particularly when sudden head movement, limited procedural tolerance, or anxiety related to holding the electrosurgical return electrode could compromise safe use of the electrosurgical device. In all cases, the surgical objective was to limit the release to the restrictive mucosal and fascial components, avoid adjacent ductal, vascular, and neural structures, and minimize unnecessary thermal or mechanical injury to adjacent tissues.

The term “lingual frenotomy” is used in this manuscript to describe a limited surgical release of the restrictive lingual frenulum and associated mucosal/fascial components, without tissue excision, flap advancement, sutured reconstruction, or muscle incision. The wound was left unsutured and allowed to heal by secondary intention. Hemostasis was achieved by local compression with gauze and, when electrosurgical incision was used, by the cutting effect of the device without additional coagulation. Postoperative recommendations included routine oral-hygiene instructions, early gentle tongue-mobility exercises, and age-appropriate analgesics when needed.

Postoperative management followed a standardized core protocol in all patients. On the day of surgery, each patient and/or caregiver received oral, written, and demonstrative instructions from the operating clinician, who was also the first author. In pediatric cases, the parent or legal guardian was instructed directly. The immediate postoperative protocol included early tongue-mobility exercises, initiated approximately 6 h after surgery, and gentle wound-margin manual procedures during the first postoperative month. The same core postoperative sequence was recommended for all patients, regardless of whether they had undergone preoperative preparation.

Approximately 4 weeks after surgery, patients and/or caregivers attended an in-office instruction visit for the home phase of the MRT-based instrument-assisted rehabilitation protocol. In this study, the term MRT refers specifically to the intraoral, instrument-assisted protocol developed by the first author and should not be conflated with conventional extraoral myofascial release approaches described in the musculoskeletal rehabilitation literature. The MRT protocol used in this study involved low-load, controlled mobilization of the sublingual and perioral soft tissues using a dedicated intraoral instrument. During this visit, the operating clinician demonstrated the technique directly on the patient using the clinical demonstration version of the instrument, which allowed the recommended pressure range and tissue-handling technique to be shown before home use. The parent/caregiver or adult patient then performed the technique under supervision to verify correct placement, direction of movement, tissue handling, and patient tolerance. An instructional video was also provided as a reminder for home use.

The recommended force range and the method for recognizing an appropriate tissue load were explained during this visit. The clinical version of the instrument allowed controlled pressure application within a range of 0.5–1.5 N/mm^2^ and was used for instruction and calibration. The home version of the instrument did not include force feedback and was intended for self-therapy according to the technique demonstrated in the clinic. Therefore, controlled pressure application was available only during in-office instruction and technique verification, whereas the actual pressure applied during home-based self-therapy could not be objectively recorded or standardized. During follow-up appointments, patients and/or caregivers were asked to demonstrate the home procedure again, allowing the technique to be verified, tissue tolerance to be reassessed, and the recommended force range to be adjusted when appropriate. Further information on the intervention, including preparation before surgery, manual procedures, successive MRT phases, neurolinguistic coordination, and nutritional guidance, is presented in Supplementary File S1.

In cases of coexisting functional disorders, including an altered breathing pattern, swallowing difficulties, or masseter muscle tension, the MFS protocol [17,18,19] was introduced to support functional normalization. Patients with oral or mixed breathing received MFS-assisted therapy using individually selected stimulators: an oral obturator, intended to improve orbicularis oris tone and reduce mouth breathing, and/or a nasal stimulator. The nasal appliance was selected to facilitate airflow through the nose by supporting nostril patency. Patients were instructed to wear the prescribed stimulators at night and for additional periods during daytime.

Written and verbal instructions regarding stimulator use and home exercises were provided to patients and/or their guardians during therapeutic consultations.

### 2.5. Follow-Up Schedule and Adherence Classification

Assessments were scheduled at five time points: V0, qualifying preoperative visit; V1, immediately after surgery; and V2–V4, subsequent postoperative assessments. The median postoperative intervals were 1.4 months for V2, 9.4 months for V3, and 15.3 months for V4, with interquartile ranges presented in Table 2.

Patient adherence to the therapeutic protocol was also evaluated. To examine the influence of adherence, patients were retrospectively stratified into adherence subgroups based on the frequency of home MRT sessions documented in consecutive follow-up records, using patient/caregiver reports together with in-office confirmation of correct technique. Patients were classified as having high adherence if they performed at least three MRT sessions per week on average during the intervals between follow-up visits. Patients were classified as having low adherence if they performed one or fewer sessions per week on average, did not initiate or continue the home protocol, or discontinued therapy entirely for at least two consecutive weeks within a follow-up interval. In the final analyzed dataset, all patients were assigned to one of these two exploratory adherence categories; no separate intermediate-adherence group was included in the final subgroup analysis. Therapeutic adherence was assessed during the postoperative follow-up period and was therefore treated as a follow-up behavioral exposure rather than a baseline characteristic.

These cutoffs were predefined pragmatic clinical thresholds rather than validated adherence categories. The threshold of ≥3 sessions per week was selected to reflect the recommended minimum frequency considered necessary to maintain repeated soft-tissue stimulation during postoperative healing, whereas ≤1 session per week was considered to represent insufficient therapeutic engagement for the purposes of this exploratory subgroup comparison. In the absence of a formal control group, this stratification enabled an exploratory internal comparison of outcomes according to therapeutic engagement. No electronic monitoring system was used to objectively record the frequency, duration, or pressure of home-based MRT sessions.

### 2.6. Statistical Analysis

Statistical analysis was performed using Statistica v.13.3 (TIBCO Software Inc., Palo Alto, CA, USA) and R version 4.4.3. All tests were two-sided, and the significance level was set at alpha = 0.05.

To account for the longitudinal structure of the IOPI data and the correlation between repeated measurements within the same patient, an expanded linear mixed-effects model was fitted using all available IOPI observations. The model included patient-specific random intercepts and fixed effects for visit, adherence category, surgical technique, age group, sex, preoperative preparation, Coryllos classification, and breathing type. Age was included as a dichotomous variable using the median age of 10 years as the cut-off. The model was specified as: IOPI ~ Visit + Adherence + Technique + Age + Sex + Preoperative preparation + Coryllos classification + Breathing type + (1 | Patient ID). This approach allowed incomplete follow-up data to be included without restricting the analysis to patients who completed the final V4 visit. The mixed-effects model was fitted in R using the lme4/lmerTest framework, with Satterthwaite’s method used to estimate degrees of freedom for fixed-effect tests.

Because TEMMO is an inverse ordinal scale, TEMMO outcomes were analyzed primarily as ordinal data. Changes in TEMMO grades across visits were assessed using the Friedman test for repeated ordinal measurements. When the Friedman test indicated a significant overall difference, post hoc pairwise comparisons between visits were performed using Conover’s test with multiplicity adjustment. TEMMO grade distributions at each visit were reported descriptively as counts and percentages. Mean and standard deviation values were retained only as descriptive summaries to allow comparison with the previous version of the analysis, whereas interpretation was based primarily on grade distributions and ordinal/non-parametric analysis.

Paired and visit-specific comparisons were retained as supportive descriptive analyses. For continuous outcomes, including maximal tongue pressure, the normality of paired differences between baseline (V0) and the final follow-up visit (V4) was assessed using the Shapiro–Wilk test. Within-group changes were analyzed using a paired *t*-test when the normality assumption was met; otherwise, the Wilcoxon signed-rank test was used. For paired V0–V4 comparisons, the standardized effect size was calculated as the standardized mean change for paired observations (Cohen’s dz), defined as the mean within-subject difference divided by the standard deviation of the within-subject differences. Effect sizes for paired comparisons were calculated only for patients with available data at both time points.

Between-group differences according to adherence level at individual visits were assessed using Welch’s *t*-test for independent samples. Missing data, particularly at V4, were explored descriptively using complementary approaches. IOPI outcomes were summarized using all available observations, last-observation-carried-forward (LOCF) imputation, and a complete-case analysis including patients with available V4 data. These analyses were used as sensitivity and descriptive robustness checks rather than as methods intended to eliminate missing-data bias.

Because of the retrospective design, the available sample size was determined by the number of eligible patients who met the inclusion criteria during the study period. A post hoc reference sample size calculation was performed for contextual purposes for the primary endpoint, defined as the within-subject change in maximal tongue pressure between baseline and the final visit. Using a paired *t*-test framework, an alpha level of 0.05, and 80% power, a sample of 64 patients would be sufficient to detect a moderate standardized within-subject effect size of Cohen’s d = 0.5. This threshold was selected as a conventional benchmark for a moderate effect size rather than being derived from previous MRT-specific pilot data.

### 2.7. Ethical Considerations

This study was conducted in accordance with the Declaration of Helsinki. Because the study involved retrospective analysis of anonymized clinical records collected during routine clinical care between 2021 and 2024, ethics approval was obtained prior to manuscript preparation rather than prior to data collection. The Bioethics Committee reviewed and approved the retrospective use of these anonymized records (approval No. 03/BNR/2025; approval date: 9 July 2025) and waived the requirement for individual informed consent for retrospective data analysis, in accordance with applicable national regulations. Written consent to publish the clinical photographs was obtained from each patient shown in the manuscript or, where applicable, from the patient’s legal guardian.

Clinical trial registration was not required because this was a retrospective observational longitudinal study.

## 3. Results

Data from the clinical records of patients treated between 2021 and 2024 were retrospectively analyzed. The inter-rater agreement for TEMMO grading was excellent (Cohen’s kappa = 0.942; 95% CI, 0.890 to 0.995). The baseline characteristics of the study group are presented in Table 1.

A total of 64 patients were included. Their ages ranged from 5 to 46 years, with a mean age of 11.7 ± 7.0 years. According to the Coryllos classification [21], the most prevalent lingual frenulum type was type 3 (78.1%). The most frequently used surgical instrument was scissors (53.1%), followed by electrosurgical incision without coagulation (45.3%). In one patient (1.6%), both techniques were used during the same procedure.

At baseline, 31.2% of patients showed asymmetry of tongue-tip elevation during maximal mouth opening. The predominant breathing pattern was nasal breathing (50.0%), although a substantial proportion of patients exhibited oral or mixed breathing. At baseline, the mean maximal tongue pressure measured with the IOPI was 35.4 ± 13.5 kPa, and the mean TEMMO score was 3.1 ± 0.4, consistent with restricted tongue mobility. By the final follow-up visit (V4), the mean tongue pressure had increased to 49.0 ± 10.2 kPa, while the mean TEMMO score had improved to 1.4 ± 0.6, indicating a marked improvement in tongue elevation.

Out of the 64 included patients, 30 (46.9%) attended the final long-term follow-up visit (V4), whereas 34 patients did not have available V4 data. Because this was a retrospective analysis of routine clinical records, reasons for missing final follow-up data were not systematically captured for all patients. No postoperative complications were documented in clinical records. To assess potential attrition bias, baseline characteristics were compared between patients who completed the V4 visit (*n* = 30) and those who did not (*n* = 34). V4 completers had higher baseline IOPI values than non-completers (median [Q1; Q3], 38 [30; 50] kPa vs. 31 [23; 41] kPa; *p* = 0.025). The surgical instrument also differed between groups (*p* = 0.023), with electrosurgical incision being more frequent among V4 completers and scissors being more frequent among non-completers. Age showed a trend toward higher values among completers (median, 12 [8; 13] years vs. 9 [7; 11] years; *p* = 0.062). No statistically significant baseline differences were observed for sex, breathing pattern, baseline TEMMO grade, Coryllos classification, preoperative preparation, or adherence category. This comparison is presented in Supplementary Table S4.

For the primary endpoint, detailed IOPI estimates are presented in Supplementary Tables S1–S3. In the analysis of all available observations, mean IOPI increased from 35.4 ± 13.5 kPa at V0 to 49.0 ± 10.2 kPa at V4 among patients with available V4 data. In the LOCF sensitivity analysis including all 64 patients, mean IOPI increased from 35.4 ± 13.5 kPa at V0 to 45.6 ± 9.7 kPa at V4, with the median increasing from 35 [24; 47] to 45 [40; 51] kPa. In the complete-case analysis including patients with complete V0–V4 data (*n* = 30), mean IOPI increased from 39.2 ± 13.6 kPa at V0 to 49.0 ± 10.2 kPa at V4, with the median increasing from 38 [30; 50] to 50 [43; 53] kPa. These analyses supported the same direction of change but should be interpreted cautiously because imputation and complete-case approaches cannot eliminate the risk of attrition bias.

### 3.1. Changes in Tongue Strength, Tongue Mobility, and Mouth Opening over Time

The primary endpoint was maximal tongue pressure measured with the Iowa Oral Performance Instrument (IOPI). Mean maximal tongue pressure increased over time, with a statistically significant improvement observed between baseline and the final follow-up visit. Because the baseline-to-final follow-up comparison included only patients with available V4 data, this result should be interpreted as a completers-only analysis.

At baseline (V0), mean tongue strength was 35.4 ± 13.5 kPa; by the final follow-up visit (V4), approximately 15 months later, it had increased to 49.0 ± 10.2 kPa. The baseline-to-V4 comparison for the primary endpoint (IOPI) was based on patients with available V4 data (*n* = 30) and should therefore be interpreted as a completers-only analysis. Sensitivity and descriptive robustness analyses using all available observations, LOCF imputation, and complete-case analysis supported the same direction of change. Detailed IOPI estimates for these analyses are presented in Supplementary Tables S1–S3. In the complete-case analysis including patients with complete V0–V4 data (*n* = 30), mean IOPI increased from 39.2 ± 13.6 kPa at V0 to 49.0 ± 10.2 kPa at V4. In the LOCF sensitivity analysis including all 64 patients, mean IOPI increased from 35.4 ± 13.5 kPa at V0 to 45.6 ± 9.7 kPa at V4. These analyses should be interpreted cautiously because neither imputation nor complete-case analysis eliminates the risk of attrition bias. The V0-to-V4 complete-case change corresponded to a large paired-samples standardized mean change (Cohen’s dz = 1.14; 95% CI, 0.75 to 1.53). A parallel improvement was observed for tongue elevation during maximum mouth opening, with the mean TEMMO decreasing from 3.1 at baseline to 1.4 at V4, consistent with improved tongue elevation range. In contrast, the range of maximum mouth opening remained relatively stable over time. Maximum mouth-opening values showed little variation across the follow-up visits. Table 2 summarizes the longitudinal changes in the principal functional outcomes.

One patient did not attend the V3 follow-up visit for logistical reasons, reducing the sample size at that time point from 64 to 63. The proportion of patients demonstrating high therapeutic adherence ranged from 51.6% to 60.0% across visits. The greatest numerical changes in the recorded functional parameters were observed between V1 and V3.

The number of completed visits was closely associated with the duration of follow-up after frenotomy (Spearman’s rho = 0.927; *p* < 0.001). This strong association indicates that in this patient group, both variables reflected closely related aspects of treatment exposure and should not be interpreted as independent factors when considering clinical outcomes.

### 3.2. Association Between Adherence and Tongue-Pressure Outcomes

In the comparative analysis of tongue strength (IOPI) between patients classified into the high and low therapeutic adherence, no statistically significant differences were observed at earlier time points. In the full study sample, 34 patients were classified as high-adherence and 30 as low-adherence. A significant between-group difference emerged only at the final follow-up visit (V4; *n* = 30 completers), as shown in Table 3. At V4, patients in the high-adherence group reached a mean tongue pressure of 52.7 kPa, whereas those in the low-adherence group reached 43.5 kPa, with a mean between-group difference of 9.2 kPa (95% CI, 0.8 to 17.6; *p* = 0.032). This finding indicates an association between consistent therapy participation and better final tongue-pressure outcomes among V4 completers (Cohen’s d = 0.66, medium-to-large effect).

In the expanded repeated-measures linear mixed-effects model for IOPI, visit remained the main factor associated with tongue-pressure values. Compared with baseline (V0), IOPI values were not significantly different immediately after surgery (V1; estimate, 0.66 kPa; 95% CI, −1.47 to 2.79; *p* = 0.543), but were significantly higher at V2 (estimate, 4.43 kPa; 95% CI, 2.30 to 6.56; *p* < 0.001), V3 (estimate, 8.59 kPa; 95% CI, 6.46 to 10.72; *p* < 0.001), and V4 (estimate, 11.17 kPa; 95% CI, 8.41 to 13.93; *p* < 0.001). Age > 10 years was also significantly associated with higher IOPI values (estimate, 5.87 kPa; 95% CI, 0.04 to 11.70; *p* = 0.048). In contrast, adherence category, surgical technique, sex, preoperative preparation, Coryllos classification, and breathing type were not independently associated with IOPI in this adjusted repeated-measures model.

### 3.3. Tongue-Mobility Outcomes

Analysis of tongue mobility assessed with TEMMO showed consistent improvement over time. Because TEMMO is an inverse ordinal scale, changes in tongue mobility were evaluated primarily using grade distributions and non-parametric repeated-measures analysis. The percentage of patients with normal tongue function (TEMMO grade 1) increased from 0% at V0 to 66.7% at V4.

The Friedman test confirmed a statistically significant change in TEMMO grades over time (χ^2^ = 189.4; df = 4; *p* < 0.001; Kendall’s W = 0.751), indicating a large time-related effect. Post hoc Conover pairwise comparisons with multiplicity adjustment showed no statistically significant difference between V0 and V1, whereas significant differences were observed from V2 onward.

The distribution of TEMMO grades showed a progressive shift toward lower TEMMO grades, indicating better tongue elevation over time. At baseline, 55 of 64 patients (85.9%) were classified as TEMMO grade 3, 8 (12.5%) as grade 4, and 1 (1.6%) as grade 5. By V4, among 30 patients with available long-term follow-up, 20 patients (66.7%) had achieved TEMMO grade 1, 6 (20.0%) had grade 2, and 4 (13.3%) had grade 3, with no patients remaining in grades 4 or 5. The median TEMMO grade [Q1; Q3] improved from 3 [3; 3] at baseline to 1 [1; 2] at V4. The full distribution of TEMMO grades at each visit is presented in Supplementary Table S5 and Supplementary Figure S1.

### 3.4. Representative Clinical Cases

**Figure 2:**
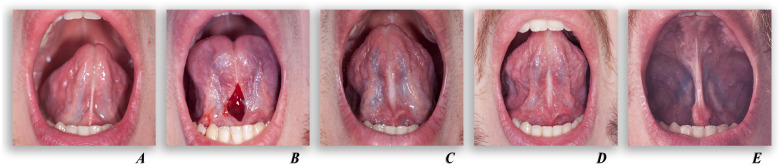
Tongue morphology and mobility at successive observation points in a 20-year-old patient who maintained full therapeutic adherence. Photographic documentation extended from the surgical visit to the 8-month follow-up after frenotomy and MRT-based fascial therapy. Time points: (**A**) preoperative presentation; (**B**) immediately after surgery; (**C**) 2 months postoperatively; (**D**) 5 months postoperatively; and (**E**) 8 months postoperatively, with complete tongue elevation and no compensatory perioral muscle activity.

**Figure 3:**
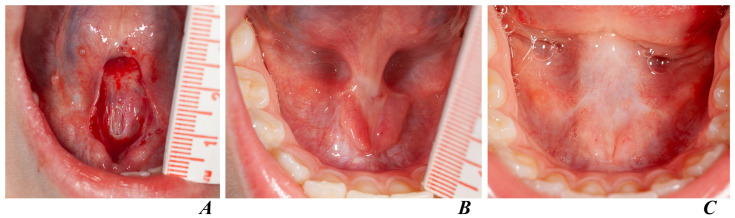
Sequential changes in the floor of the mouth in a 10-year-old patient. Time points: (**A**) immediately after surgical release; (**B**) 1 month postoperatively, showing scar formation; and (**C**) after 18 months of MRT therapy, demonstrating further expansion of the floor-of-the-mouth soft-tissue area.

**Figure 4:**
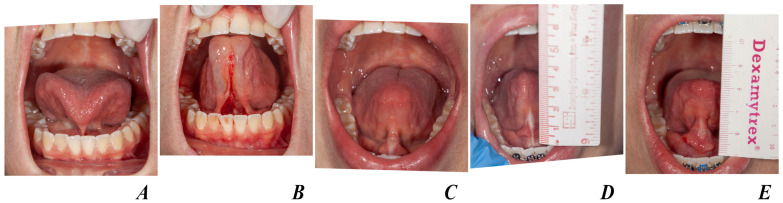
Tongue-mobility findings at successive time points in a 14-year-old female patient who received no preoperative preparation and showed limited therapeutic adherence. The observation period extended from surgery to the 15-month follow-up. Time points: (**A**) restricted tongue elevation before surgery; (**B**) appearance immediately after the procedure; and (**C**–**E**) follow-up clinical photographs obtained at maximum mouth opening. The later photographs indicate that functional improvement was only partly maintained in the absence of regular MRT therapy.

Among the 64 patients included in the study, selected clinical cases are presented in Figure 2, Figure 3 and Figure 4 to illustrate changes in tongue morphology and function in patients with different levels of therapeutic adherence, as well as the variability in treatment response following frenotomy combined with the MRT protocol. These cases are presented for illustrative purposes only and were not used as independent evidence of tissue remodeling, vascular changes, histological adaptation, or treatment efficacy. Clinical photographs were used to document visible functional and morphological changes during follow-up, but no histological, vascular, or biomechanical tissue assessment was performed. These cases illustrate the potential influence of preoperative preparation, therapeutic adherence, and individual anatomical features on treatment course and outcome.

#### 3.4.1. Case 1: High-Adherence Patient

Functional changes in this fully adherent 20-year-old patient are illustrated over the course of treatment. The photograph obtained on the surgical day, following MRT-based fascial preparation, showed leftward deviation of the tongue associated with a right-sided crossbite (Figure 2A). Relative to the original diagnostic assessment, preoperative therapy had already produced a moderate improvement in tongue elevation and midline control, with TEMMO changing from grade 3 to an intermediate clinical value between grades 2 and 3.

The immediate postoperative image (Figure 2B) was obtained after the electrosurgical frenotomy performed in incision mode without coagulation, using two incisions: one between the alveolar ridge and the sublingual duct and the second extending from the duct to the ventral frenulum insertion. At 2 months, early improvement in tongue elevation and midline control was observed (Figure 2C). At 5 months, further improvement in symmetry of muscle activity during vertical tongue elevation was noted (Figure 2D). By 8 months, the patient demonstrated active palatal suction, full elevation of the tongue tip, and no visible compensatory perioral activity (Figure 2E).

All images were obtained during active elevation of the tongue tip toward the incisive papilla with the mouth maximally open. Over the course of follow-up, tongue mobility improved from TEMMO grade 3 to grade 1 and tongue pressure increased from 61.7 to 68.3 kPa. The greatest functional improvement was observed between 2 and 8 months, with complete tongue elevation and absence of compensatory muscle activity at the final follow-up.

#### 3.4.2. Case 2: High-Adherence Patient

The clinical photographs document the floor-of-the-mouth appearance in a 10-year-old patient at three successive stages. The first photograph was obtained immediately after fascial release performed by electrosurgical incision without coagulation (Figure 3A), 1 month postoperatively with visible scar formation (Figure 3B), and after 18 months of manual therapy using the MRT (Figure 3C). The immediate postoperative image suggests the initial extent of tissue release; however, at 1 month, partial secondary restriction was visible, consistent with the early healing phase.

By the end of long-term MRT therapy (Figure 3C), the images show an apparent increase in the visible soft-tissue area in the floor of the mouth compared with the 1-month follow-up. The final image also shows a different tissue surface appearance; however, vascular or histological changes cannot be inferred from clinical photographs alone and were not assessed in this study.

Overall, the serial images illustrate visible postoperative changes in the floor-of-mouth soft tissues and tongue mobility during follow-up. However, the underlying biological processes responsible for these clinical changes cannot be determined from photographs alone.

#### 3.4.3. Case 3: Low-Adherence Patient

This 14-year-old female patient received no preoperative preparation and showed limited adherence during follow-up. Before surgery, tongue elevation was markedly restricted (TEMMO grade 4; Coryllos type 1 frenulum attachment; Figure 4A). An immediate increase in the available tongue-elevation range was observed after frenotomy (Figure 4B). Electrosurgery was used in incision mode without coagulation. Two limited incision lines were made: the first between the alveolar ridge and the sublingual ductal region, and the second between the ductal region and the ventral frenulum attachment. A multilayered fascial structure was observed intraoperatively. Clinical photographs obtained at maximum mouth opening during the subsequent 15 months showed that the initial functional change was only partially maintained (Figure 4C–E).

This case illustrates a limited functional response in the context of irregular postoperative MRT therapy. TEMMO changed from grade 4 to grade 3, representing an estimated gain of approximately 10 mm in tongue-elevation range. IOPI pressure values showed only a small change, increasing from 50.3 to 52.0 kPa.

Despite reporting that tongue mobility felt better, the patient showed limited motivation to continue the prescribed home protocol. Inadequate postoperative stimulation may have contributed to suboptimal soft-tissue adaptation and secondary restriction of fascial elasticity; however, this cannot be confirmed using clinical photographs alone. Despite reporting that tongue mobility felt better, the patient showed limited motivation to continue the prescribed home protocol.

In this case, frenotomy had been recommended for orthodontic reasons, including anterior open bite and maxillary dental crowding, whereas the patient and caregivers had not previously recognized tongue-mobility limitation. This may have reduced adherence to the recommended postoperative protocol.

## 4. Discussion

The retrospective design limited full control over potentially relevant variables. Nevertheless, the longitudinal follow-up, objective outcome measures, and serial clinical observations provide clinically meaningful information on functional change after lingual frenotomy combined with MRT-based therapy. These findings suggest that postoperative outcomes may depend not only on the surgical release itself but also on the quality and continuity of structured therapeutic follow-up, including myofascial rehabilitation, such as the MRT protocol used in this study.

Importantly, in the adjusted repeated-measures model, visit remained significantly associated with IOPI improvement, whereas adherence category and surgical technique were not independently associated with IOPI. Therefore, the adherence-related findings from visit-specific comparisons should be interpreted as exploratory and hypothesis-generating rather than as evidence of an independent causal effect of adherence.

The biological rationale for MRT is based primarily on the mechanosensitivity of connective and fascial tissues rather than on mucosal healing alone. Fascia is a collagen-rich, mechanically responsive tissue in which fibroblasts and myofibroblasts participate in extracellular-matrix remodeling, collagen turnover, and changes in tissue stiffness and gliding properties. Preclinical studies of instrument-assisted soft-tissue mobilization have suggested that controlled mechanical loading may influence fibroblast activity and collagen repair in injured tendon models. However, these findings provide only indirect support for MRT, because tendon, skin, oral mucosa, and sublingual fascial tissues differ substantially in structure, vascularity, innervation, and healing behavior. Therefore, the present findings should be interpreted as clinical longitudinal observations, whereas any proposed fibroblast-, collagen-, or fascia-mediated mechanisms remain hypothesis-generating and require direct investigation in sublingual and oral fascial tissues [22,23,24]. Collagen turnover and the balance between collagen synthesis and degradation may influence extracellular-matrix organization and tissue biomechanics during healing [25]. Anatomical studies have shown considerable inter-individual variation in the structure of the lingual and floor-of-mouth fascia, including differences in fascial layering, collagen composition, and midline thickening, all of which may influence tissue biomechanics and response to treatment [6,7]. In this context, the prolonged course of recovery observed in some patients may be consistent with the gradual nature of connective-tissue remodeling [22,23]. The observed functional improvement in patients of different ages suggests that this therapeutic approach may have clinical utility across developmental stages, although this should be interpreted cautiously because of the limited number of adult participants. Mechanical stimuli applied to fascial tissues may influence fibroblast and myofibroblast activity, extracellular-matrix remodeling, collagen deposition, and fascial mechanical properties, including stiffness and tissue gliding. Because fascia is mechanically integrated with muscle, such changes may also modify the mechanical environment of adjacent muscles [26]. Further mechanistic studies are needed to clarify whether MRT is associated with measurable changes in sublingual connective-tissue remodeling, collagen turnover, and the temporal balance between type I and type III collagen during postoperative healing.

The chosen frequency of MRT exercises, once every 24 h, was based on the intended spacing of mechanical stimulation over time and on broader concepts of mechanically induced soft-tissue adaptation described in the rehabilitation literature [24]. In addition to its theoretical rationale, a once-daily schedule with brief sessions was considered practical and may have supported better adherence in routine clinical use.

The therapeutic tool used in this study was designed to deliver controlled pressure in the range of 0.5 to 1.5 N/mm^2^. Experimental studies in tendon tissue have suggested that pressure magnitude may influence fibroblast proliferation; however, these findings were obtained in rat Achilles tendon and cannot be directly extrapolated to floor-of-mouth tissues, which differ substantially in structure and mechanical behavior [10,13]. In routine clinical use, most patients, particularly children, perceived 0.5 N/mm^2^ as the highest pressure that remained tolerable, especially at the beginning of therapy. Higher pressures were often reported as uncomfortable in the early stages. With time, some patients were able to tolerate gradual increases in pressure, and the applied intensity could be adjusted during follow-up visits according to individual tolerance. In this clinical sample, a pressure of approximately 0.5 N/mm^2^ appeared to be clinically acceptable and feasible for repeated use. Whether oral soft tissues respond biologically to relatively low levels of mechanical stimulation remains unclear and requires direct investigation in future studies.

Beyond the question of mechanical dose, the present findings also highlight the importance of clinical decision-making before and after frenotomy. In routine practice, some patients may be referred for surgical release primarily on the basis of anatomical appearance, despite relatively good functional tongue mobility. In the present clinical setting, patients referred for suspected ankyloglossia were therefore re-evaluated using a functional assessment approach, including TEMMO grading, breathing pattern, lip competence and resting lip posture, signs of lip dryness or chapping, and relevant developmental and functional history. Frenotomy was not pursued when tongue mobility was normal or borderline and the overall functional assessment did not support a clear surgical indication.

This broader diagnostic approach is important because restricted tongue function may reflect not only the anatomical characteristics of the lingual frenulum but also coexisting functional disorders, including oral or mixed breathing, altered lip competence, and abnormal oral motor patterns. Conversely, in patients who do undergo frenotomy, immediate postoperative improvement in tongue elevation may not necessarily be maintained during the early healing phase. Scar formation, fascial stiffness, altered motor habits, and insufficient postoperative stimulation may contribute to secondary functional restriction. This clinical problem provided the rationale for using a structured MRT-based postoperative protocol in the present study, with the aim of supporting soft-tissue adaptation and functional re-education rather than simply increasing the extent of surgical release.

The need for such a functional perspective is further supported by the asymmetry findings observed in the study group. Asymmetry of tongue-tip elevation during maximal mouth opening was observed in 31.2% of patients, and representative cases included unilateral crossbite. Although this relationship was not formally tested in the present study, these observations may suggest a broader pattern of transverse functional imbalance. This interpretation is consistent with previous studies suggesting that muscle tone and functional asymmetries within the oral cavity may affect craniofacial skeletal and dental arch development [27,28]. Beyond its possible influence on fascial tissues, MRT may also exert effects related to the anatomical region in which it is applied. The richly innervated sublingual tissues and ventral aspect of the tongue may constitute relevant sites for sensory stimulation.

It can be hypothesized that pressure applied with the MRT tool stimulates sensory or proprioceptive receptors within floor-of-mouth musculature, with afferent input potentially influencing trigeminal motor circuits and thereby contributing to functional re-education of tongue posture and masticatory muscle tone [29]. These mechanisms were not directly investigated in this study and should therefore be regarded as biologically plausible but speculative.

The IOPI is a reliable tool for measuring tongue strength [20]. Objective measurement of tongue force is relevant in understanding the functional properties of the tongue, as force production depends on biomechanical conditions, including muscle length [30]. In the present study, tongue strength increased over time, particularly in patients who adhered to the MRT protocol, suggesting that functional improvement after frenotomy was not limited to mobility alone but also involved progressive gains in force-generating capacity. Reduced tongue strength may reflect not only anatomical restriction but also impaired functional use. It may therefore be hypothesized that structured postoperative rehabilitation contributes to improved tongue performance through progressive soft-tissue adaptation over time. However, the biological mechanisms underlying this process were not directly assessed.

Importantly, periods of major change in tongue size and strength overlap with recognized phases of craniofacial growth, including infancy, childhood, and puberty [1,31]. There is increasing evidence that tongue function is relevant to dentofacial development. The maxilla and mandible develop through growth and remodeling processes that are responsive to mechanical influences from surrounding soft tissues, including the tongue, in keeping with the functional matrix concept [32,33,34]. Although, in this study, we did not directly assess skeletal outcomes, the observed increase in tongue strength may be clinically relevant from an orthodontic perspective, particularly during active developmental stages.

From a developmental perspective, tissue formation begins in the first months of fetal life, when the architecture of craniofacial growth zones, including the mandible, is already being established. Mechanical forces such as tension, compression, and muscular resistance may contribute to the differentiation of mesenchymal cells and to the formation of mineralized and chondroid tissues involved in membranous bone development and growth [32,35,36]. It can therefore be hypothesized that forces generated by the tongue are relevant modulators of craniofacial adaptation, particularly during periods of intense remodeling associated with dental eruption and developmental peaks in tongue size and strength [1,31].

Accordingly, tongue mobility, resting posture, muscle tone, and contact with the palate may contribute to the functional environment in which the facial skeleton develops. A stable tongue posture, palatal contact, and nasal breathing may also support coordinated tongue motor functional force generation. These functional conditions could influence neuromechanical signaling within craniofacial soft tissues. Therapeutic management may therefore aim to improve the balance of muscular forces, support fascial organization, and promote tongue mobility together with nasal breathing. In the present study, higher tongue-pressure values were observed during follow-up after frenotomy and adjunctive MRT. Although this finding may be compatible with adaptive soft-tissue remodeling, the underlying mechanotransduction mechanisms were not directly investigated.

The orthodontic relevance of our findings is supported by previous studies linking oral muscle pressure and tongue force to malocclusion and dentofacial morphology. Children with malocclusion in the mixed dentition have been shown to exhibit altered oral muscle pressure patterns compared with controls, highlighting the importance of functional diagnosis in orthodontic prevention, treatment, and stability [37]. Lower posterior tongue pressure has also been associated with shorter ramus height, reduced posterior facial height, and clockwise mandibular rotation, suggesting a relationship between tongue pressure and a more vertical skeletal pattern [38]. A systematic review of myofunctional and orthodontic treatment in patients with atypical swallowing concluded that tongue position is associated with malocclusion and that several therapies show promise in correcting this relationship [39]. Tongue position has also been shown to influence masseter and temporalis activity during maximal voluntary clenching, supporting a functional link between intraoral sensorimotor conditions and masticatory muscle behavior [40].

### Limitations

This study has several important limitations. First, the retrospective observational design and the absence of a formal control group substantially limit causal interpretation. All patients underwent lingual frenotomy followed by postoperative rehabilitation; therefore, the observed longitudinal changes cannot be attributed specifically to surgery, MRT-based rehabilitation, postoperative exercises, natural healing, growth, maturation, motor learning, or concomitant interventions alone. The present findings should therefore be interpreted as exploratory longitudinal clinical observations rather than as evidence of treatment efficacy. Prospective controlled studies using ethically appropriate comparator groups are needed to determine the relative contribution of each therapeutic component, for example by comparing standardized postoperative care with or without predefined adjunctive rehabilitation modules.

Second, age should also be considered when interpreting tongue-pressure outcomes. Although age was included as a covariate in the adjusted mixed-effects model and was significantly associated with IOPI values, the study was not sufficiently powered for detailed age-stratified analyses, particularly because only a small number of adult participants were included. Growth, maturation, orthodontic development, and motor learning may therefore have contributed to the longitudinal increase in tongue pressure, especially in children and adolescents. Future studies should include larger age-stratified samples and should interpret IOPI outcomes in relation to age-specific normative values where available.

Third, because the study was referral-based, selection bias cannot be excluded. In addition, outcome assessment was not blinded, which may have introduced observer bias, particularly for functional clinical evaluations. Although the core postoperative instruction protocol was standardized and delivered by the same operating clinician, variability in how patients or caregivers performed and maintained the home exercises could not be fully controlled and may have introduced performance bias.

Fourth, follow-up completion was incomplete, with only 30 of 64 patients attending the final V4 visit. This substantial attrition may have introduced bias, particularly because V4 completers differed from non-completers in baseline IOPI values and surgical instrument distribution. Therefore, the long-term V4 estimates may overrepresent patients with better baseline tongue strength, different treatment characteristics, greater motivation, better access to care, or greater perceived benefit. LOCF and complete-case analyses were used only as sensitivity and descriptive robustness checks and should not be interpreted as eliminating missing-data bias. This is particularly important in a pediatric and adolescent sample, because tongue pressure may change over time because of growth, maturation, orthodontic development, and motor learning, independently of the intervention.

Fifth, adherence was assessed retrospectively from clinical records, patient/caregiver reports, and in-office confirmation of technique. No electronic monitoring system was used to objectively record the frequency, duration, quality, or pressure of home-based MRT sessions. The adherence categories were pragmatic clinical thresholds rather than validated adherence measures. In addition, adherence was a post-baseline behavioral exposure and may have been influenced by perceived improvement, motivation, family involvement, logistical barriers, and follow-up attendance. Therefore, the observed association between higher adherence and better final IOPI values among V4 completers should be interpreted as exploratory and hypothesis-generating rather than as evidence of an independent causal effect of adherence. Concomitant speech-language therapy and the exact use of individual MFS-related appliances, including oral obturators and nasal stimulators, were not systematically coded as separate analyzable variables in the statistical dataset; therefore, their independent effects could not be isolated or included as separate covariates in the multivariable model.

Sixth, several co-interventions may have influenced the observed outcomes. Some patients received preoperative manual or MRT-based preparation, concomitant speech–language therapy, MFS-related functional interventions, oral obturators, nasal stimulators, or orthodontic-related care. Because these interventions were part of individualized routine clinical management, their independent effects could not be isolated. Residual confounding by co-interventions, baseline functional status, breathing pattern, occlusal characteristics, and follow-up duration cannot be excluded.

Seventh, although an expanded repeated-measures mixed-effects model was added to account for within-patient correlation and to adjust for visit, adherence category, surgical technique, age group, sex, preoperative preparation, Coryllos classification, and breathing type, the model still did not fully adjust for all potentially relevant confounders. Concomitant speech-language therapy and the exact use of individual MFS-related appliances, including oral obturators and nasal stimulators, were not systematically coded as separate analyzable variables and therefore could not be included as independent covariates. In addition, baseline IOPI and TEMMO were not included as separate covariates in this repeated-measures framework, and detailed follow-up duration was not modeled separately because it was closely related to visit number. Therefore, residual confounding by unmodeled baseline functional differences, co-interventions, motivation, access to care, and follow-up-related factors cannot be excluded.

Eighth, TEMMO is an inverse ordinal scale, and although categorical distributions and non-parametric repeated-measures analyses were added, mean values were retained only as descriptive summaries. Half-grade values recorded in routine clinical notes were not treated as separate validated TEMMO categories. This reflects the retrospective clinical nature of the dataset and should be considered when interpreting tongue-mobility outcomes.

Ninth, the biological mechanisms underlying the observed functional changes were not directly investigated. The discussion of connective-tissue remodeling, fascial mechanosensitivity, fibroblast activity, collagen turnover, and mechanotransduction provides only biological plausibility and should be interpreted as hypothesis-generating. No histological, vascular, biomechanical, imaging-based, or molecular assessment of sublingual tissues was performed. Therefore, clinical photographs and functional measurements cannot establish the underlying tissue-level mechanisms responsible for the observed changes.

Finally, the first author developed the MRT protocol and designed the dedicated intraoral instruments used in this therapeutic approach, and intellectual property rights are related to the MRT instrument. Although the statistical analysis was performed independently and the interpretation has been made conservative, this potential conflict of interest should be considered when interpreting the findings. Future studies should also evaluate the role of breathing-pattern modification in patients with restricted tongue mobility, not only after lingual frenotomy but also in broader clinical contexts in which tongue elevation is functionally limited. Prospective protocols should assess whether a shift from oral or mixed breathing toward nasal breathing is associated with changes in TEMMO grade, resting tongue posture, lip competence, and intraoral pressure balance. Such studies would help determine whether improvement in the functional breathing environment contributes to changes in tongue mobility independently of, or in addition to, surgical release, wound healing, myofascial rehabilitation, growth, and motor learning.

Future independent, prospective, controlled studies with blinded outcome assessment, standardized adherence monitoring, predefined follow-up schedules, and larger multicenter samples are required to establish the clinical role of MRT as an adjunct to lingual frenotomy.

## 5. Conclusions

In this retrospective observational longitudinal study, patients undergoing lingual frenotomy followed by adjunctive MRT-based rehabilitation showed favorable longitudinal changes in objective tongue-function measures, including tongue mobility, assessed using TEMMO, and maximal tongue pressure, assessed using the IOPI. Higher therapeutic adherence was associated with better long-term tongue-pressure outcomes among patients who completed the final follow-up. These findings may be consistent with a potential role for structured postoperative functional rehabilitation in supporting tongue-function outcomes after lingual frenotomy. However, because of the retrospective design, absence of comparator groups, incomplete long-term follow-up, and the presence of concomitant interventions, these findings should be interpreted as exploratory and hypothesis-generating rather than as evidence of a causal treatment effect. Prospective controlled studies with standardized intervention arms and objective adherence monitoring are needed to confirm these observations and clarify the relative contributions of surgery, rehabilitation, natural development, and adherence. Future studies should also evaluate whether MRT-based therapy may have a role as a standalone approach in selected borderline cases with equivocal indications for frenotomy. The preoperative improvement observed in some patients suggests that this question may be clinically relevant; however, this remains a hypothesis-generating observation requiring prospective evaluation.

## Figures and Tables

**Table 1:** Baseline characteristics of 64 patients at the first visit (V0).

Variable	Summary
Sex, no. (%)	Female 35 (54.7); male 29 (45.3)
Age, years	Mean 11.7 (SD 7.0); median 10 (8–13); range 5–46
Breathing pattern, no. (%)	Nasal 32 (50.0); oral 12 (18.8); mixed 20 (31.2)
Tongue strength by IOPI, kPa	Mean 35.4 (SD 13.5); median 35 (24–47); range 10.3–67.0
Maximal mouth opening, mm	Mean 48.8 (SD 4.5); median 50 (45–51); range 40–64
TEMMO grade	Mean 3.1 (SD 0.4); median 3 (3–3); range 3–5
Tongue deviation at maximal mouth opening, no. (%)	Absent 35 (54.7); left 16 (25.0); right 13 (20.3)
Coryllos frenulum type, no. (%)	Type 1, 4 (6.2); type 2, 9 (14.1); type 3, 50 (78.1); type 4, 1 (1.6)
Instrument used for frenotomy, no. (%)	Scissors 34 (53.1); electrosurgical incision without coagulation 29 (45.3); both techniques 1 (1.6)
Preoperative preparation, no. (%)	None 33 (51.6); manual exercises 8 (12.5); myofascial release technique 23 (35.9)

Note: percentages refer to mutually exclusive categories based on the primary instrument used; one patient received both techniques during the same procedure. IOPI—Iowa Oral Performance Instrument; TEMMO—Tongue Elevation at Maximal Mouth Opening.

**Table 2:** Changes in selected clinical parameters over time and the proportion of patients demonstrating high therapeutic adherence at each study visit (V0–V4).

Visit	N	Time (Months), Median [Q1; Q3]	IOPI (kPa), Mean ± SD	TEMMO Grade, Mean ± SD	RMMO (mm), Median [Q1; Q3]	High Adherence, *n* (%)
V0	64	-	35.4 ± 13.5	3.1 ± 0.4	50 [45; 51]	-
V1	64	0	36.0 ± 12.7	2.9 ± 0.6	50 [45; 51]	34 (53.1%)
V2	64	1.4 [1.1; 2.6]	39.8 ± 11.7	2.3 ± 0.7	49 [45; 53]	33 (51.6%)
V3	63	9.4 [6.1; 13.1]	44.1 ± 9.4	1.7 ± 0.8	50 [47; 53]	34 (54.0%)
V4	30	15.3 [12.0; 29.5]	49.0 ± 10.2	1.4 ± 0.6	50 [49; 52]	18 (60.0%)

Note: IOPI, Iowa Oral Performance Instrument; TEMMO, Tongue Elevation at Maximal Mouth Opening; RMMO, range of maximum mouth opening. Adherence is reported as a follow-up behavioral exposure and was not treated as a baseline characteristic.

**Table 3:** Mean tongue-pressure values (IOPI, kPa) in patient groups stratified by therapeutic adherence across successive study visits.

Visit Number (Follow-Up), Median [Q1; Q3]	High-Adherence, Mean ± SD (*n*)	Low-Adherence, Mean ± SD (*n*)	*p*-Value
V0: pre-surgery	34.7 ± 15.1 (*n* = 34)	36.1 ± 11.8 (*n* = 30)	0.684
V1: 0 months	36.0 ± 13.7 (*n* = 34)	36.1 ± 11.6 (*n* = 30)	0.973
V2: 1.4 [1.1; 2.6] months	39.3 ± 11.7 (*n* = 34)	40.4 ± 11.9 (*n* = 30)	0.722
V3: 9.4 [6.1; 13.1] months	45.5 ± 8.3 (*n* = 34)	42.4 ± 10.5 (*n* = 29)	0.202
V4: 15.3 [12.0; 29.5] months	52.7 ± 6.7 (*n* = 18)	43.5 ± 12.2 (*n* = 12)	0.032
V0 versus V4	*p* = 0.001	*p* = 0.009	

Note: Values are presented as mean ± SD, with row-specific sample sizes shown in parentheses. *p*-values for between-group comparisons were calculated using Welch’s *t*-test for independent samples. Within-group V0 versus V4 comparisons were calculated using paired *t*-tests within each adherence group and included only patients with available data at both V0 and V4.

## Data Availability

The data are not publicly available because they originate from retrospective clinical records and contain patient-level clinical information. Anonymized derived data can be made available from the corresponding author upon reasonable request and subject to applicable ethical and privacy requirements.

## Supplementary Materials

The following supporting information can be downloaded at https://www.mdpi.com/article/10.3390/jcm15135171/s1, Supplementary File S1: Therapeutic management protocol and speech–language therapy integration after lingual frenotomy with MRT; Supplementary Tables S1–S4: Additional IOPI analyses and attrition assessment; Supplementary Table S5: Distribution of TEMMO grades across study visits; Supplementary Table S6: STROBE-based checklist for a retrospective observational longitudinal study; Supplementary Figure S1: Home-Based Intraoral Stimulation Protocol; Supplementary Figure S2: Lingual nerve location in neonatal and adult anatomical material; Supplementary Figure S3: STROBE-style flow diagram.

## Abbreviations

Abbreviation	Definition
IASTM	Instrument-Assisted Soft-Tissue Mobilization
IOPI	Iowa Oral Performance Instrument
MFS	Multifunctional System
MRT	Myofascial Release Technique
RMMO	Range of maximum mouth opening
TEMMO	Tongue Elevation at Maximal Mouth Opening
