# Supplementary material for: Objective Tongue-Function Outcomes After Lingual Frenotomy with Adjunctive Myofascial Rehabilitation: A Retrospective Observational Longitudinal Study
Source: J Clin Med. 2026 Jul 2;15(13):5171. doi: 10.3390/jcm15135171 (PMC13363322; doi:10.3390/jcm15135171)
Supplement: Supplementary file 1 [file jcm-15-05171-s001.zip › STROBE_MRT_observational_longitudinal_revised_final.pdf]

STROBE-based Checklist and Flow Diagram

Objective Tongue-Function Outcomes after Lingual Frenotomy with Adjunctive Myofascial Rehabilitation: A Retrospective Observational Longitudinal Study

Study design: Retrospective observational longitudinal study using anonymized routine clinical records.

Checklist scope: Adapted from the STROBE recommendations for observational studies. Wording was aligned with a retrospective longitudinal design and avoids terminology that could imply a prospective comparative exposure-group design.

Supplementary Table S6. STROBE-based checklist for a retrospective observational longitudinal study

Section	Item	STROBE recommendation / adapted wording	Reported?	Location / relevant text in manuscript
Title and abstract	1a	Indicate the study design with a commonly used term in the title or abstract.	Yes	The title identifies the article as a Retrospective Observational Longitudinal Study; the abstract also states the retrospective observational longitudinal design. Location: Title page; Abstract.
Title and abstract	1b	Provide an informative and balanced summary of what was done and what was found.	Yes	The abstract summarizes background, data source, participants, clinical protocol, IOPI/TEMMO outcomes, adherence classification, adverse-event assessment, and cautious conclusions. Location: Abstract.
Introduction	2	Explain the scientific background and rationale for the investigation.	Yes	The introduction describes ankyloglossia, objective functional assessment, postoperative soft-tissue healing, IASTM/MRT rationale, and the need for objective longitudinal outcome data. Location: Introduction.
Introduction	3	State specific objectives, including any prespecified hypotheses.	Yes	The primary aim was to describe longitudinal changes in tongue mobility and maximal tongue pressure after lingual frenotomy followed by adjunctive myofascial rehabilitation; the secondary exploratory objective concerned adherence. Location: End of Introduction.
Methods	4	Present key elements of study design early in the paper.	Yes	Section 2.1 identifies the work as a retrospective observational longitudinal analysis of anonymized clinical records collected during routine care. Location: Methods 2.1.
Methods	5	Describe the setting, locations, and relevant dates, including periods of treatment, follow-up, and data collection.	Yes	Records were collected from a private dental practice providing orthodontic and myofunctional care in Poland during routine care between 2021 and 2024; follow-up visits V0-V4 are described. Location: Methods 2.1 and 2.5.
Methods	6a	Give eligibility criteria, sources and methods of participant selection, and methods of follow-up.	Yes	Eligible patients were 5-46 years old, had restricted tongue mobility documented by TEMMO at qualifying assessment, underwent lingual frenotomy, and had baseline/follow-up IOPI and TEMMO assessments. Follow-up visits V0-V4 are described. Location: Methods 2.1 and 2.5.
Methods	6b	For matched or grouped observational analyses, give matching or grouping criteria where applicable.	Not applicable	This study did not use matching and did not include predefined matched exposed and unexposed groups. Location: Not applicable.
Methods	7	Clearly define outcomes, clinical exposure/context, predictors, potential confounders, and effect modifiers.	Yes	Primary outcome: maximal tongue pressure measured by IOPI. Secondary outcomes: TEMMO grade distribution, grade 1 achievement, RMMO, and adverse events. Clinical context: lingual frenotomy with adjunctive MRT-based rehabilitation. Exploratory adherence categories and covariates are defined. Location: Methods 2.1-2.6.

Section	Item	STROBE recommendation / adapted wording	Reported?	Location / relevant text in manuscript
Methods	8	For each variable, give data sources and details of measurement/assessment methods.	Yes	IOPI, TEMMO, RMMO, adverse-event record review, adherence assessment, surgical technique, breathing pattern, and preoperative preparation are described. TEMMO inter-rater reliability is reported. Location: Methods 2.2-2.6.
Methods	9	Describe efforts to address potential sources of bias.	Yes	The revised manuscript addresses retrospective design, absence of a comparator group, attrition bias, subjective adherence assessment, non-systematically coded co-interventions, age/growth effects, unblinded assessment, and potential conflict of interest. Location: Methods 2.6; Results attrition analysis; Discussion/Limitations.
Methods	10	Explain how the study size was arrived at.	Yes	The study size was determined by all eligible records during the study period; a post hoc reference sample-size calculation is provided for the primary endpoint. Location: Statistical Analysis 2.6.
Methods	11	Explain how quantitative variables were handled in the analyses; describe groupings and rationale.	Yes	IOPI and RMMO were summarized as continuous variables; TEMMO was treated primarily as an inverse ordinal variable with grade distributions. Age was dichotomized at the median of 10 years; adherence thresholds were pragmatic clinical categories. Location: Methods 2.2, 2.5, and 2.6.
Methods	12a	Describe statistical methods, including methods used to control for confounding.	Yes	An expanded linear mixed-effects model for IOPI used patient-specific random intercepts and fixed effects for visit, adherence, technique, age, sex, preoperative preparation, Coryllos classification, and breathing type; TEMMO was assessed with non-parametric repeated-measures analysis. Location: Statistical Analysis 2.6.
Methods	12b	Describe methods used to examine subgroups and interactions.	Yes/limited	Exploratory subgroup comparisons by adherence category were performed. More detailed subgroup analyses were limited by sample size; this is acknowledged. Location: Methods 2.5-2.6; Results 3.2; Limitations.
Methods	12c	Explain how missing data were addressed.	Yes	Missing V4 data were handled descriptively by all-available observations, LOCF sensitivity analysis, complete-case analysis, and mixed-effects modeling; limitations of these approaches are acknowledged. Location: Methods 2.6; Results; Limitations.
Methods	12d	For longitudinal observational studies, explain how incomplete follow-up was addressed.	Yes	The number of patients at each visit is reported; Patients with available V4 data and those without V4 data are compared at baseline; V4 is interpreted as a long-term analysis based on patients with available V4 data. Location: Results; Supplementary Table S4.
Methods	12e	Describe any sensitivity analyses.	Yes	LOCF sensitivity analysis, complete-case analysis, all-available observation summaries, and non-parametric/ordinal analysis for TEMMO are described. Location: Methods 2.6; Results 3.1 and 3.3; Supplementary Tables S1-S4.
Results	13a	Report numbers of individuals at each stage: eligible, included, follow-up, and analyzed.	Yes	The manuscript reports 64 patients at baseline, 64 at V1, 64 at V2, 63 at V3, and 30 at V4. A STROBE-style flow diagram is included in this document. Location: Results 3.1/Table 2; Flow diagram.
Results	13b	Give reasons for non-participation or incomplete follow-up at each stage, where available.	Partially	The flow diagram reports the number of patients without V4 data (n = 34). Because this was a retrospective analysis of routine clinical records, reasons for missing final follow-up data were not systematically captured for all patients; this limitation and the potential for attrition/missing-data bias are acknowledged. Location: Results; Flow diagram note; Limitations.
Results	13c	Consider use of a flow diagram.	Yes	A STROBE-style flow diagram summarizes eligible records and visit-level follow-up. Location: Flow diagram in this document.

Section	Item	STROBE recommendation / adapted wording	Reported?	Location / relevant text in manuscript
Results	14a	Give characteristics of study participants and information on relevant clinical variables and potential confounders.	Yes	Baseline characteristics include sex, age, breathing type, IOPI, RMMO, TEMMO, tongue deviation, Coryllos type, surgical tool, and preoperative preparation. Location: Results/Table 1.
Results	14b	Indicate the number of participants with missing data for each variable of interest.	Yes	Visit-level availability is described; V4 was available for 30/64 patients. Missing-data handling and sensitivity summaries are reported. Location: Results 3.1; Methods 2.6; Supplementary Tables.
Results	14c	Summarize follow-up time when relevant.	Yes	Follow-up visits are described as V0, V1, V2, V3, and V4; median follow-up intervals are reported where available. Location: Methods 2.5; Results 3.1/Table 2.
Results	15	Report outcome data or summary measures over time.	Yes	IOPI, TEMMO, RMMO, and adverse-event outcomes are reported across visits. TEMMO distributions and non-parametric analyses are provided in supplementary materials. Location: Results 3.1-3.3; Tables/Figures; Supplementary Tables.
Results	16a	Give unadjusted estimates and, where applicable, confounder-adjusted estimates with precision.	Yes	Descriptive longitudinal estimates, adherence-group comparisons, confidence intervals for key comparisons, and an adjusted repeated-measures model for IOPI are reported. Location: Results 3.1-3.3; Tables 2-4; Supplementary Tables.
Results	16b	Report category boundaries when continuous variables were categorized.	Yes	Age was dichotomized at 10 years; adherence was categorized as high versus low according to pragmatic clinical thresholds. Location: Methods 2.5-2.6.
Results	16c	If relevant, translate relative risk into absolute risk.	Not applicable	The study did not estimate relative risks for incident events; outcomes were continuous/ordinal functional measures and adverse events were reported descriptively. Location: Not applicable.
Results	17	Report other analyses, including subgroup, interaction, and sensitivity analyses.	Yes	Adherence subgroup analyses, mixed-effects modeling, LOCF/complete-case sensitivity summaries, TEMMO non-parametric analysis, and comparison between patients with and without V4 data are reported. Location: Results 3.1-3.3; Supplementary Tables S1-S4.
Discussion	18	Summarize key results with reference to study objectives.	Yes	The Discussion summarizes longitudinal changes in IOPI and TEMMO, adherence-related findings, attrition and limitations, and clinical interpretation. Location: Discussion.
Discussion	19	Discuss limitations, including sources of bias or imprecision.	Yes	Limitations include retrospective design, no comparator group, attrition bias, missing-data limitations, heterogeneity, co-interventions, subjective adherence, lack of objective home monitoring, limited adult subgroup, ordinal TEMMO issues, mechanistic uncertainty, and potential conflict of interest. Location: Limitations.
Discussion	20	Give cautious overall interpretation considering objectives, limitations, multiplicity, similar studies, and evidence.	Yes	The interpretation is framed as exploratory and hypothesis-generating; the manuscript avoids causal claims and states that controlled prospective studies are required. Location: Discussion and Conclusions.
Discussion	21	Discuss generalizability/external validity.	Yes	The manuscript acknowledges that the sample came from a referral-based, predominantly pediatric, single-practice routine-care setting, with only a small adult subgroup, limiting generalizability. Location: Discussion/Limitations.
Other information	22	Give source of funding and role of funders.	Yes	The manuscript states that the research received no external funding. Conflicts of interest and instrument-related intellectual property are disclosed. Location: Funding and Conflicts of Interest.
Other information	Ethics/consent	Describe ethics approval and consent process.	Yes	Ethics approval was obtained for retrospective analysis of anonymized clinical records, with waiver of prospective individual informed consent. Separate written consent was obtained for

Section	Item	STROBE recommendation / adapted wording	Reported?	Location / relevant text in manuscript
				publication of clinical photographs. Location: Ethical Considerations 2.7; IRB/Informed Consent statements.

Supplementary Figure S3. STROBE-style flow diagram

Supplementary Figure S3. STROBE-style flow diagram showing the number of eligible clinical records included at baseline and the number of patients with available follow-up data at each study visit.

Clinical records screened from routine care records
Treated in a private dental practice in Poland, 2021-2024

↓

Eligible records included at baseline (V0)
n = 64

↓

Immediate postoperative assessment (V1)
Available data: n = 64

↓

Early follow-up assessment (V2)
Available data: n = 64

↓

Intermediate follow-up assessment (V3)
Available data: n = 63

↓

Long-term follow-up assessment (V4)
Available data: n = 30

↓

Patients without V4 data
n = 34; final long-term follow-up measurements unavailable

Flow diagram note: Because this was a retrospective analysis of routine clinical records, a complete prospective screening log of all non-included individuals was not available. V4 findings were therefore interpreted as long-term analyses based on available V4 data, supported by all-available-observation summaries and sensitivity analyses.
