# Supplementary material for: Objective Tongue-Function Outcomes After Lingual Frenotomy with Adjunctive Myofascial Rehabilitation: A Retrospective Observational Longitudinal Study
Source: J Clin Med. 2026 Jul 2;15(13):5171. doi: 10.3390/jcm15135171 (PMC13363322; doi:10.3390/jcm15135171)
Supplement: Supplementary file 1 [file jcm-15-05171-s001.zip › Supplementary File S1.pdf]

Supplementary File S1

Therapeutic Management Protocol and Speech–Language Therapy Integration after Lingual Frenotomy with Myofascial Release Technique (MRT)

This supplementary file describes the clinical pathway used for patients undergoing lingual frenotomy followed by adjunctive myofascial rehabilitation. The protocol combined a standardized core postoperative pathway with individualized preoperative preparation and force/tolerance adjustment. Individualization was based on Tongue Elevation at Maximal Mouth Opening (TEMMO) grade, treatment stage, tissue tolerance, anatomical restriction, and caregiver/patient cooperation.

S1.1. Protocol Overview

The therapeutic pathway combined two complementary components: low-load manual preparation and tool-assisted MRT. Depending on TEMMO grade, treatment stage, and tissue tolerance, management could include preoperative manual preparation, preoperative MRT tool-based preparation, early postoperative mobility exercises, and subsequent home-based tool-assisted intraoral stimulation.

S1.2. Preoperative Management According to TEMMO Grade

S1.2.1. Patients with TEMMO Grade 3

In patients with TEMMO grade 3, preoperative therapy could be initiated using the MRT instrument after prior in-office instruction with the clinical demonstration version. Pressure selection was individualized during follow-up visits within a range of 0.5-1.5 N/mm², depending on patient tolerance. In routine clinical practice, the most commonly tolerated pressure was 0.5 N/mm². Tolerance was monitored during subsequent visits, and gradual force escalation was introduced when appropriate. The home-use sequence and force-selection principles were otherwise analogous to those described below for the postoperative MRT tool protocol after day 30.

S1.2.2. Patients with TEMMO Grades 4 or 5

In patients with TEMMO grades 4 or 5, low-load intraoral and extraoral manual preparation was recommended before tool-assisted therapy or surgery. These techniques were performed by the therapist or caregiver without the use of the instrument. Exercises likely to induce excessive tension in the region of the lingual frenulum attached to the gingiva were avoided.

S1.3. Objectives for the Intraoral Manual and MRT Tool-Assisted Techniques

The objectives were as follows:

- To reduce myofascial tension in the floor of the mouth and tongue;
- To improve vertical tongue mobility;
- To increase tolerance to sublingual contact and intraoral tissue mobilization;
- To optimize biomechanical conditions for tongue mobility assessment and subsequent therapy.

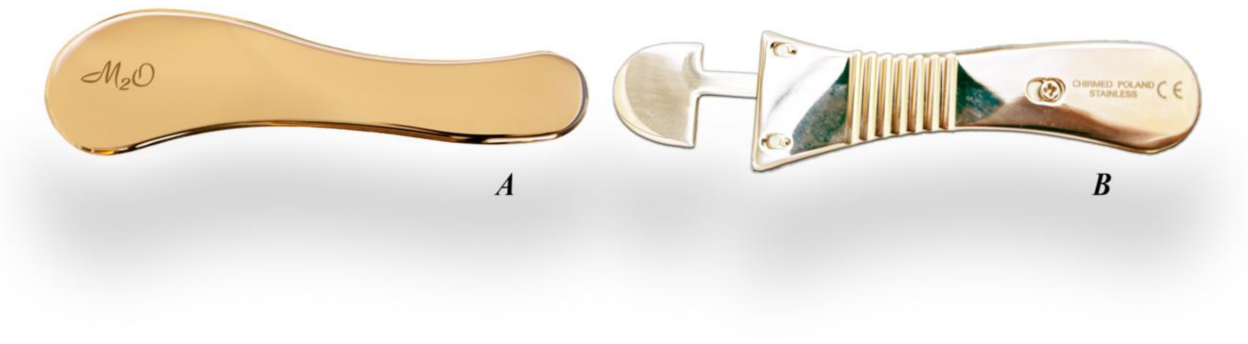

Figure S1.1. MRT instruments used in the therapeutic protocol. (A) Simplified stainless-steel MRT instrument designed for home use. (B) Clinical version equipped with an internal system for controlled pressure application (0.5-1.5 N/mm²), used for educational and monitoring purposes.

S1.4. General Principles of Manual and Tool-Assisted MRT

All techniques were performed slowly and gently, without pain and without forceful stretching of the frenulum. The patient was positioned upright with stable support during therapy. The aim was to promote soft-tissue relaxation, improve tolerance to touch and mobilization, and reduce protective tone in the floor-of-mouth and suprahyoid regions.

For manual preparation, gentle tissue stretching or sustained low-load contact was initially maintained for 30 seconds and progressively increased to 60-120 seconds depending on tolerance.

For tool-assisted therapy, the pressure level was first established during in-office instruction with the clinical demonstration instrument. The most commonly tolerated initial pressure in routine clinical use was approximately 0.5 N/mm², particularly in children and in the early phases of therapy. The force used at home was based on the individual level accepted during instruction and was subsequently reviewed during follow-up visits.

S1.5. Intraoral Manual Techniques

Intraoral techniques included unilateral and bilateral sublingual contact.

For unilateral sublingual contact, the therapist placed the index finger under the tongue on the left side of the inferior attachment region and maintained gentle, low-load contact pressure for 30 seconds, progressing to 60-120 seconds as tolerated. The same procedure was then repeated on the right side. Within a session, the dose was introduced as one repetition per side and gradually increased up to five repetitions per side, with approximately 5-10 seconds of rest between repetitions.

For bilateral sublingual contact, two index fingers were placed intraorally on both sides of the inferior attachment region and gentle pressure was maintained for 30-60 seconds. When tolerated, the fingers were positioned deeper under the tongue bilaterally. After approximately 30 seconds of low-load contact, gentle elevation of the ventral surface of the tongue in its midportion was applied without traction on the frenulum. For bilateral holds, the dose was introduced as one repetition per session and gradually increased up to five repetitions per session, with both sides treated simultaneously and approximately 5-10 seconds of rest allowed between repetitions.

During selected unilateral intraoral holds, a two-point contact technique could be used, combining intraoral sublingual contact with the index finger of one hand and simultaneous extraoral support under the mandible with the fingers of the other hand. This maneuver was intended to stabilize the tissues by approximating the two points of contact.

A **B** **C**

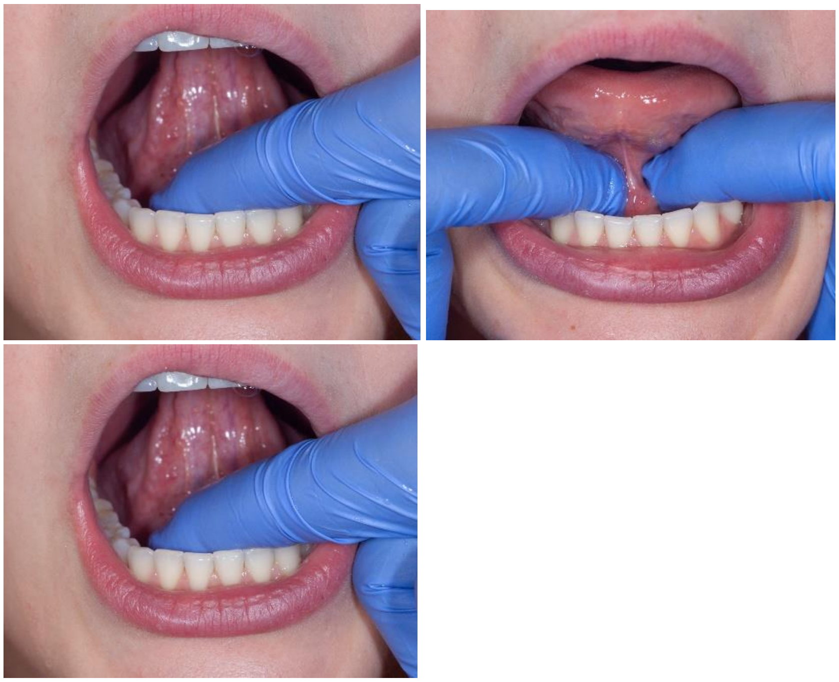

Figure S1.2. Representative examples of intraoral manual preparation techniques used before or after frenotomy: (A) unilateral sublingual contact, (B) intraoral contact during guided tongue mobilization, and (C) bilateral sublingual contact. The image has been cropped to limit patient identifiability.

S1.6. Extraoral Techniques

Extraoral techniques included submental holds and slow gliding strokes. For the submental hold, the thumb or index finger was placed under the chin, just below the mental tubercle, and maintained for 30-60 seconds. For slow submental gliding, two fingers were placed in the submental region and a very slow gliding movement was performed toward the mandibular angle. This maneuver was repeated 3-5 times.

S1.7. Delivery of Manual Preparation

Manual preparation was typically delivered as supervised sessions by a speech–language therapist over approximately 1 month preoperatively, usually once weekly for a total of approximately four sessions.

Between visits, patients and caregivers were instructed to perform the same low-load intraoral and extraoral procedures at home.

S1.8. Management From Day 0 to Day 30 After the Procedure

On the day of surgery, the patient and/or caregiver received oral, written, and demonstrative instructions from the operating clinician. In pediatric cases, the parent or legal guardian was instructed directly. The day 0–30 protocol formed the standardized core postoperative pathway and was recommended for all patients after frenotomy, regardless of whether preoperative preparation had been performed.

S1.8.1. Mobility Exercises

Mobility exercises were performed once daily. Each movement was initiated with 2-3 repetitions, depending on patient tolerance. After 1 week, the protocol was progressed to three sets of five repetitions per exercise.

- The patient was instructed to attempt to touch the tip of the tongue to the second molar in the upper and lower arch on each side while keeping the mouth open as wide as possible, using a mirror for visual feedback.
- The patient was instructed to touch the tip of the tongue to the buccal mucosa on each side while maintaining maximal mouth opening and avoiding mandibular movement, using a mirror for visual feedback.
- The patient was instructed to suction the tongue to the palate and then open and close the mouth without detaching the tongue.

S1.8.2. Early Intraoral Wound-Margin Manual Therapy

Intraoral manual therapy was performed by the parent or caregiver four times daily.

Using a gloved index finger or sterile gauze, gentle-to-moderate sustained pressure was applied directly to the right and left lateral wound margins, that is, the two lateral edges of the incision site. Pressure was directed medially toward the center of the wound without scraping. The pressure was maintained for 2-3 seconds on each side and repeated 3-5 times.

For posterior displacement of the tongue tip, a gloved finger or sterile gauze was placed on the dorsal surface of the anterior tongue tip, approximately the anterior 1-2 cm. Gentle-to-moderate pressure was applied while, simultaneously, the tongue tip was guided posteriorly in a straight line, without upward lifting. The maneuver was maintained for 2-3 seconds and repeated 3-5 times.

A finger was gently inserted between the alveolar process and the lateral aspect of the tongue while the tongue was elevated.

S1.9. Postoperative MRT Tool Protocol After Day 30

From day 30 after the procedure, home use of the MRT instrument was recommended as the main tool-assisted component of postoperative therapy.

S1.9.1. Initial Instruction Phase

The initial phase involved supervised instruction in the use of the instrument with the clinical demonstration version. Patients and/or caregivers were instructed in the home use of the MRT tool and technique, including how to perform intraoral mobilization and how to recognize and apply the force

considered appropriate for the tissues. During this instruction process, pressure levels corresponding to 0.5, 1.0, and 1.5 N/mm² were demonstrated to the patient and/or caregiver to facilitate recognition of the target pressure range during home use. The applied pressure was individually selected within a range of 0.5-1.5 N/mm² depending on patient tolerance. Force selection was guided primarily by patient tolerance: the recommended level was defined as a pressure that did not cause pain or remained at the threshold of discomfort, whereas pressures causing clear discomfort but no pain were used only when considered acceptable and well tolerated. In routine clinical practice, the most commonly tolerated initial pressure was 0.5 N/mm². After supervised in-office instruction, video instructions were provided to the patient and/or caregiver as a reminder for home use.

A **B** **C**

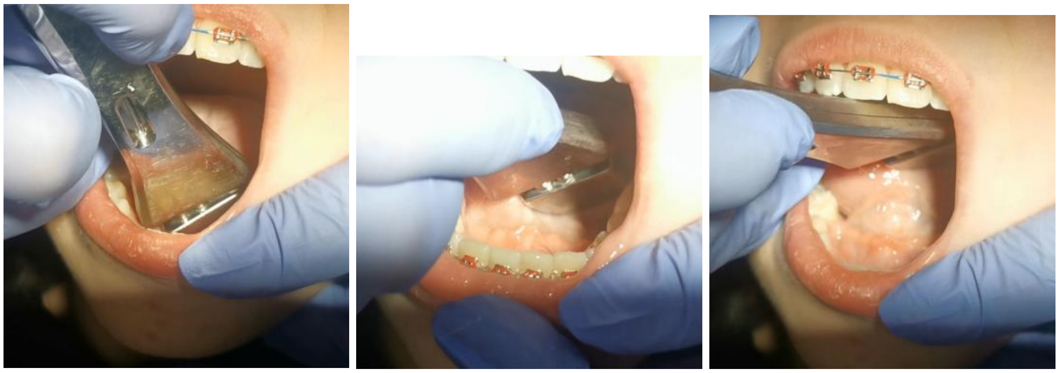

Figure S1.3. Initial in-office instruction in MRT tool use: (A) placement of the instrument immediately posterior to the alveolar process; (B,C) sliding movement along the left floor of the mouth toward the tip of the tongue, just lateral to the midline.

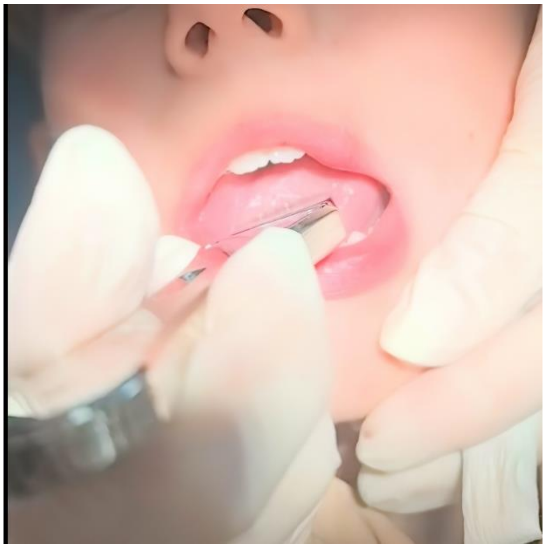

Figure S1.4. In-office instruction in the final resisted phase of the protocol. The patient performs an isometric exercise by pushing the tongue against the instrument. The image has been cropped to limit patient identifiability.

S1.9.2. Home-Based Intraoral Stimulation Protocol

At home, the protocol was performed using the home version of the MRT instrument, according to the technique demonstrated through in-office instruction. Patients and/or caregivers were advised to follow the same sequence of maneuvers and to use the pressure range established in the clinic. In cases of uncertainty, additional visits could be scheduled so that the patient or caregiver could further practice and refine the technique under supervision. In routine clinical practice, this approach appeared practical for home use because the target pressure was defined relative to a familiar sensory limit, that is, a level that did not cause pain or remained at the threshold of discomfort.

The home protocol followed a fixed sequence. First, the narrow edge of the instrument was used for sliding mobilization of the floor of the mouth on each side, from the area posterior to the alveolar process along the genioglossus region toward the tip of the tongue, just lateral to the midline. This maneuver was performed for approximately 3 minutes on the left side and 3 minutes on the right side. The sequence was then repeated using the wide side of the instrument to stimulate the central floor of the mouth and the ventral surface of the tongue on the midline for approximately 3 minutes. In the final phase, resisted stimulation of the ventral surface of the tongue was performed, followed by an isometric exercise in which the patient actively pushed the tongue against the instrument while the person performing the procedure applied gentle counterpressure by directing the instrument posteriorly toward the throat. During the last repetitions, resistance was gradually increased according to tolerance and the contraction was maintained for approximately 10 seconds.

S1.9.3. Follow-Up Calibration

During follow-up visits, the clinical version of the instrument was used to verify the correctness of the home technique, reassess tissue tolerance, and adjust the recommended force range when appropriate. Patients and/or caregivers were asked to demonstrate the home procedure, which allowed direct verification of the technique. Patients were also asked to demonstrate palatal tongue suction in the so-called jellyfish position and to open and close the mouth while keeping the tongue suctioned to the palate. When appropriate, higher pressure levels were then tested, again according to the principle that the applied force should not cause pain.

S1.10. Resting Tongue Posture and Maintenance of Positioning

The following recommendations were given:

- Lips kept closed during rest, with nasal breathing;
- Tongue in gentle contact with the palate with light suction (the jellyfish position).

S1.11. Speech–Language Therapy Integration

Speech–language therapy integration focused on the following:

- Activation of newly achieved ranges of tongue motion, including elevation, contact with the molars, and gentle palatal suction;
- Functional correction of laterality, resting posture, and swallowing;
- Articulatory preparation through elevation of a broad and relaxed tongue toward the alveolar ridge;

- /r/ therapy using alveolarization-based progression from /t/, /d/, /td/, /gd/, and related articulatory sequences;
- Automation of target phonemes in spontaneous speech.

S1.12. Additional Therapy in Selected Patients

In selected cases, therapy aimed at modifying the breathing pattern was implemented in parallel to support the normalization of intraoral pressures and maintenance of the tongue in an appropriate resting position.

Interdisciplinary collaboration with an orthodontist was undertaken in patients with coexisting malocclusion.

S1.13. Optional Nutritional Support Discussed During Routine Care

When clinically appropriate, nutritional support for tissue regeneration and soft-tissue remodeling was discussed with patients or caregivers, considering age, body weight, diet, and medical status. The following were considered:

- Vitamin C supplementation at 0.5-1 g/day;
- Coenzyme Q10 supplementation at 10-30 mg/day;
- Dietary supplementation with amino acids, including proline and hydroxyproline, from natural sources or age- and body weight-adjusted preparations.
- This nutritional support was not mandatory, was individualized according to age, body weight, diet, and medical status, and was not analyzed as an independent study variable or outcome modifier in the present retrospective study.

S1.14. Clinical Remarks and Age-Related Considerations

The analyzed cohort included patients aged 5 years and older. Therefore, the feasibility, safety, and clinical utility of MRT-based protocols in children younger than 5 years were not evaluated in the present study. In younger children with oral functional limitations, low-load manual therapy may be considered only as part of individualized clinical management and under professional supervision. Further prospective studies are needed to assess the feasibility, safety, and clinical utility of MRT-based protocols in younger age groups.
