# Supplementary material for: Objective Tongue-Function Outcomes After Lingual Frenotomy with Adjunctive Myofascial Rehabilitation: A Retrospective Observational Longitudinal Study
Source: J Clin Med. 2026 Jul 2;15(13):5171. doi: 10.3390/jcm15135171 (PMC13363322; doi:10.3390/jcm15135171)
Supplement: Supplementary file 1 [file jcm-15-05171-s001.zip › Supplementary_Figure_S2_Lingual_Nerve_Location_FINAL_REVISED.pdf]

# Supplementary Figure S2. Lingual nerve location in neonatal and adult anatomical material

Topographic relationship of lingual-nerve branches, ductal openings, and the lingual frenulum/floor-of-mouth region relevant to incision planning.

**Supplementary Figure S2A.** Neonatal specimens: lingual nerve location in the floor-of-mouth and frenulum region.

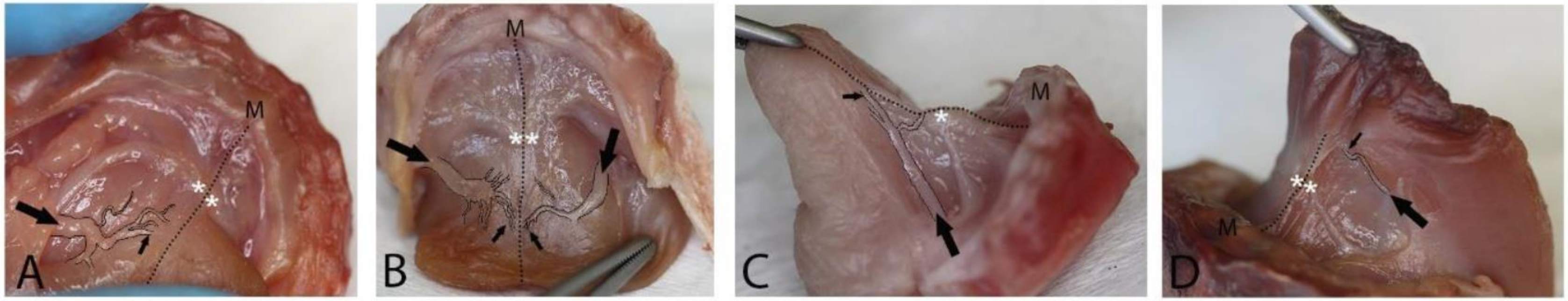


**Legend for S2A:** Black arrows indicate the lingual nerve main trunk and medial branches passing toward the frenulum region; fine black lines indicate smaller lingual-nerve branches. White stars indicate submandibular duct openings/sublingual caruncles. The black dotted line indicates the midline. M = mandible.

**Supplementary Figure S2B.** Adult specimens: lingual nerve branches on the ventral tongue after removal of the floor-of-mouth fascia.

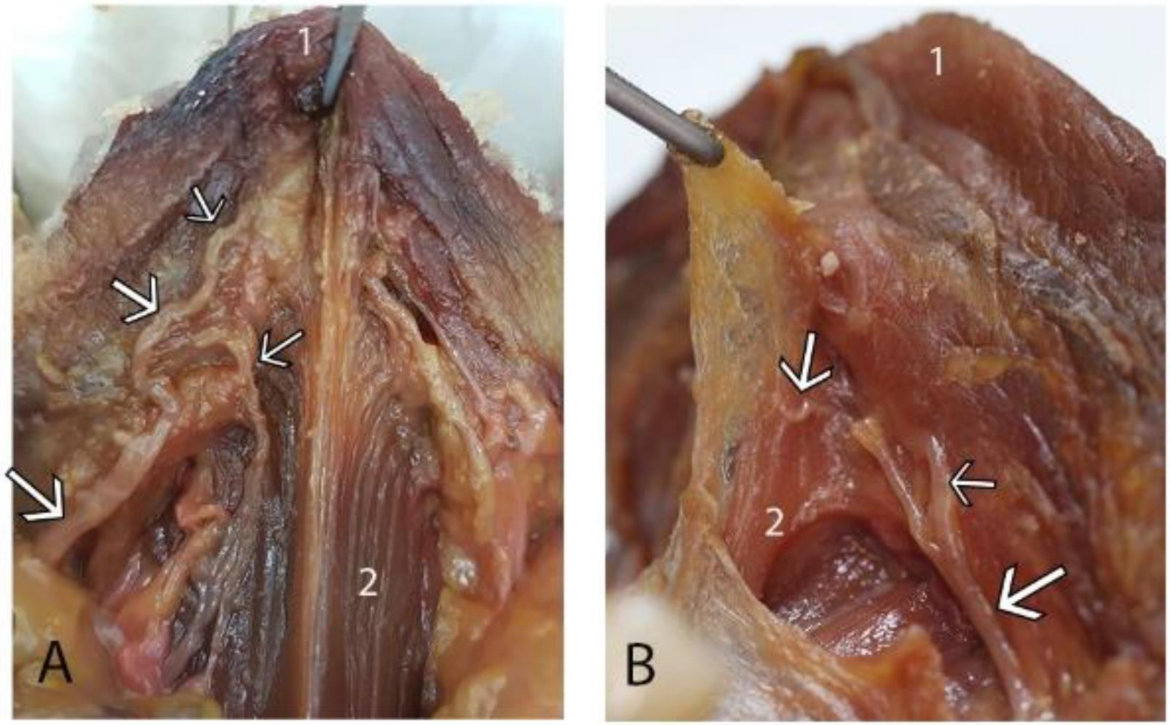


**Legend for S2B:** White arrows indicate superficial branches of the lingual nerve. 1 = ventral tongue tip; 2 = genioglossus.

### Interpretive comment and surgical relevance

The neonatal and adult anatomical material illustrates a comparable topographic relationship between the floor-of-mouth fascia, the lingual frenulum region, the submandibular duct openings/sublingual caruncles, and superficial branches of the lingual nerve. In the neonatal specimens, medial branches of the lingual nerve are shown passing toward the frenulum region in close relation to the ductal openings and midline structures. In the adult specimens, lingual-nerve branches are shown on the ventral tongue immediately beneath the fascial layer, with branches passing toward the anterior tongue and connective tissues associated with genioglossus suspension. These observations support a cautious, limited frenotomy approach rather than midline frenulum excision, wide lateral extension, deep muscular incision, or unnecessary thermal/mechanical injury in areas where superficial lingual-nerve branches may be present. In the present surgical protocol, two small incision zones were planned anterior and posterior to the submandibular duct openings/caruncles, in order to release the restrictive mucosal-fascial component while reducing the risk of lateral or deep dissection toward neural and ductal structures.

*Images courtesy of Nikki Mills; reproduced with permission.*
