# Supplementary material for: Objective Tongue-Function Outcomes After Lingual Frenotomy with Adjunctive Myofascial Rehabilitation: A Retrospective Observational Longitudinal Study
Source: J Clin Med. 2026 Jul 2;15(13):5171. doi: 10.3390/jcm15135171 (PMC13363322; doi:10.3390/jcm15135171)
Supplement: Supplementary file 1 [file jcm-15-05171-s001.zip › Supplementary_Material_S3_IOPI_attrition.pdf]

Supplementary Material S1-4

Additional IOPI analyses and attrition assessment

IOPI: Iowa Oral Performance Instrument; LOCF: last observation carried forward; V0: baseline/pre-frenotomy visit; V1: immediately post-procedure; V2-V4: postoperative follow-up visits. Values are presented as mean +/- SD, median [Q1; Q3], or n (%), as appropriate.

Table S1. Tongue strength measured with IOPI (kPa) at subsequent time points: analysis of all available results.

IOPI	V0<br>N = 64	V1<br>N = 64	V2<br>N = 64	V3<br>N = 63	V4<br>N = 30
**Mean +/- SD**	35.4 +/- 13.5	36.0 +/- 12.7	39.8 +/- 11.7	44.1 +/- 9.4	49.0 +/- 10.2
**Mdn [Q1; Q3]**	35 [24; 47]	37 [27; 45]	39 [33; 47]	43 [38; 49]	50 [43; 53]
**Min-Max**	10.3-67.0	10.3-67.0	14.0-69.3	22.0-68.3	22.0-69.0

Table S2. Tongue strength measured with IOPI (kPa) at subsequent time points: missing data handled using the last observation carried forward (LOCF) method.

IOPI	V0<br>N = 64	V1<br>N = 64	V2<br>N = 64	V3<br>N = 64	V4<br>N = 64
**Mean +/- SD**	35.4 +/- 13.5	36.0 +/- 12.7	39.8 +/- 11.7	44.0 +/- 9.4	45.6 +/- 9.7
**Mdn [Q1; Q3]**	35 [24; 47]a	37 [27; 45]a	39 [33; 47]a,b	43 [38; 49]b	45 [40; 51]b
**Min-Max**	10.3-67.0	10.3-67.0	14.0-69.3	22.0-68.3	22.0-69.0

Different superscript letters indicate statistically different post-hoc groups in the repeated-measures comparison.

Table S3. Tongue strength measured with IOPI (kPa) at subsequent time points: complete-case analysis including patients with V0-V4 data.

IOPI	V0<br>N = 30	V1<br>N = 30	V2<br>N = 30	V3<br>N = 30	V4<br>N = 30
**Mean +/- SD**	39.2 +/- 13.6	39.8 +/- 12.5	41.2 +/- 12.5	45.5 +/- 10.5	49.0 +/- 10.2
**Mdn [Q1; Q3]**	38 [30; 50]a	40 [34; 49]a	41 [35; 48]b	46 [38; 50]c	50 [43; 53]d
**Min-Max**	10.3-61.7	10.3-60.7	16.7-69.3	22.0-68.3	22.0-69.0

Different superscript letters indicate statistically different post-hoc groups in the repeated-measures comparison.

Table S4. Comparison of baseline characteristics between patients who completed the V4 visit and those who did not.

Variable	Completed N = 30	Incomplete N = 34	p-value
Female, n (%)	19 (63.3)	16 (47.1)	0.218
Age (years), Mdn [Q1; Q3]	12 [8; 13]	9 [7; 11]	0.062
**Breathing type**			0.603
Nasal, n (%)	15 (50.0)	17 (50.0)	
Oral, n (%)	7 (23.3)	5 (14.7)	
Mixed, n (%)	8 (26.7)	12 (35.3)	
Tongue strength measured with IOPI (kPa), Mdn [Q1; Q3]	38 [30; 50]	31 [23; 41]	0.025
Range of maximum mouth opening (mm), Mdn [Q1; Q3]	50 [46; 51]	48 [45; 50]	0.194
**TEMMO grade (Duran scale)**			0.492
3 deg, n (%)	26 (86.7)	29 (85.3)	
4 deg, n (%)	3 (10.0)	5 (14.7)	
5 deg, n (%)	1 (3.3)	0 (0.0)	
**Tongue deviation during maximal mouth opening**			0.396
No, n (%)	14 (46.6)	21 (61.8)	
Left, n (%)	8 (26.7)	8 (23.5)	
Right, n (%)	8 (26.7)	5 (14.7)	
**Coryllos frenulum type**			0.756
Type 1, n (%)	2 (6.7)	2 (5.9)	
Type 2, n (%)	5 (16.7)	4 (11.8)	
Type 3, n (%)	23 (76.6)	27 (79.4)	
Type 4, n (%)	0 (0.0)	1 (2.9)	
**Tool used for frenotomy**			0.023
Scissors, n (%)	11 (36.7)	23 (67.7)	
Electrosurgical incision without coagulation, n (%)	19 (63.3)	11 (32.3)	
**Preoperative preparation**			0.114
No, n (%)	17 (56.7)	16 (47.1)	
Yes, manual exercises, n (%)	1 (3.3)	7 (20.6)	
Yes, MRT, n (%)	12 (40.0)	11 (32.3)	
Low adherence, n (%)	13 (43.3)	19 (55.9)	0.453

Note: Continuous variables are presented as median [Q1; Q3]; categorical variables are presented as n (%). IOPI: Iowa Oral Performance Instrument; TEMMO: Tongue Elevation at Maximal Mouth Opening; MRT: Myofascial Release Technique.
