# Supplementary material for: Objective Tongue-Function Outcomes After Lingual Frenotomy with Adjunctive Myofascial Rehabilitation: A Retrospective Observational Longitudinal Study
Source: J Clin Med. 2026 Jul 2;15(13):5171. doi: 10.3390/jcm15135171 (PMC13363322; doi:10.3390/jcm15135171)
Supplement: Supplementary file 1 [file jcm-15-05171-s001.zip › Supplementary_Table_TEMMO_distribution.pdf]

## Supplementary Table S5. Distribution of TEMMO grades across study visits

Values are presented as n (%). TEMMO, Tongue Elevation at Maximal Mouth Opening; V0, baseline; V1, immediately after surgery; V2-V4, postoperative follow-up visits.

| **Grade** | **Time** | | | | |
|---|---|---|---|---|---|
| | **V0**<br>*n = 64* | **V1**<br>*n = 64* | **V2**<br>*n = 64* | **V3**<br>*n = 63* | **V4**<br>*n = 30* |
| 1° | 0 (0.0%) | 0 (0.0%) | 4 (6.2%) | 29 (46.0%) | 20 (66.7%) |
| 2° | 0 (0.0%) | 4 (6.2%) | 27 (42.2%) | 22 (34.9%) | 6 (20.0%) |
| 3° | 55 (85.9%) | 51 (79.7%) | 31 (48.5%) | 10 (15.9%) | 4 (13.3%) |
| 4° | 8 (12.5%) | 8 (12.5%) | 2 (3.1%) | 2 (3.2%) | 0 (0.0%) |
| 5° | 1 (1.6%) | 1 (1.6%) | 0 (0.0%) | 0 (0.0%) | 0 (0.0%) |

Note: Percentages were calculated using the number of patients available at each visit as the denominator.
